# Review on Mycotoxin Issues in Ruminants: Occurrence in Forages, Effects of Mycotoxin Ingestion on Health Status and Animal Performance and Practical Strategies to Counteract Their Negative Effects

**DOI:** 10.3390/toxins7083057

**Published:** 2015-08-12

**Authors:** Antonio Gallo, Gianluca Giuberti, Jens C. Frisvad, Terenzio Bertuzzi, Kristian F. Nielsen

**Affiliations:** 1 Institute of Feed & Food Science and Nutrition, Università Cattolica del Sacro Cuore, Piacenza 29122, Italy; E-Mails: gianluca.giuberti@unicatt.it (G.G.); terenzio.bertuzzi@unicatt.it (T.B.); 2 Department of Systems Biology, Technical University of Denmark, Building 221, Kgs. Lyngby DK-2800, Denmark; E-Mails: jcf@bio.dtu.dk (J.C.F.); kfn@bio.dtu.dk (K.F.N.)

**Keywords:** mycotoxins, silage, hay, dairy cow, heifers, ruminants

## Abstract

Ruminant diets include cereals, protein feeds, their by-products as well as hay and grass, grass/legume, whole-crop maize, small grain or sorghum silages. Furthermore, ruminants are annually or seasonally fed with grazed forage in many parts of the World. All these forages could be contaminated by several exometabolites of mycotoxigenic fungi that increase and diversify the risk of mycotoxin exposure in ruminants compared to swine and poultry that have less varied diets. Evidence suggests the greatest exposure for ruminants to some regulated mycotoxins (aflatoxins, trichothecenes, ochratoxin A, fumonisins and zearalenone) and to many other secondary metabolites produced by different species of *Alternaria* spp. (e.g., AAL toxins, alternariols, tenuazonic acid or 4Z-infectopyrone), *Aspergillus flavus* (e.g., kojic acid, cyclopiazonic acid or β-nitropropionic acid), *Aspergillus fuminatus* (e.g., gliotoxin, agroclavine, festuclavines or fumagillin), *Penicillium roqueforti* and *P. paneum* (e.g., mycophenolic acid, roquefortines, PR toxin or marcfortines) or *Monascus ruber* (citrinin and monacolins) could be mainly related to forage contamination. This review includes the knowledge of mycotoxin occurrence reported in the last 15 years, with special emphasis on mycotoxins detected in forages, and animal toxicological issues due to their ingestion. Strategies for preventing the problem of mycotoxin feed contamination under farm conditions are discussed.

## 1. Introduction

Mycotoxins are defined as molecules of low molecular weight produced by fungi that elicit a toxic response through a natural route of exposure both in humans and other vertebrate animals [1,2,3]. They are often very stable molecules and all are secondary metabolites of molds belonging to several genera, in particular *Aspergillus*, *Fusarium*, and *Penicillium* spp. [4,5,6]. Furthermore, other genera such as *Alternaria*, *Chaetomium*, *Cladosporium*, *Claviceps*, *Diplodia*, *Myrothecium*, *Monascus*, *Phoma*, *Phomopsis*, *Pithomyces*, *Trichoderma* and *Stachybotrys* include mycotoxigenic species [7,8,9,10,11]. Mycotoxin contamination represents a worldwide problem for various agricultural commodities both pre and post-harvest [7,12,13]. To date, there are about 18,000 fungal secondary metabolites described in Antibase2014, but only a restricted number [4,14] has received scientific interest from the 1960s and onwards (Table 1). As expected, the most studied are regulated mycotoxins (*i.e.*, aflatoxins (AFs), citrinin, trichothecenes such as deoxynivalenol (DON), patulin, ochratoxin A (OTA), fumonisins (FBs) and zearalenone (ZEA)) and some major toxins of endophytic fungi (ergot toxins and ergotamine).

Generally, the term mycotoxicosis refers to the syndromes resulting from ingestion, skin contact or inhalation of these fungal metabolites [1,7,15,16,17,18,19]. When livestock ingest one or more mycotoxins, the effect on health could be acute, meaning evident signs of disease are present or even causing death. However, acute manifestation of mycotoxicosis is rare under farm conditions, e.g., mainly seen in South America from *Baccharis* plants that have endophyte infection [20,21]. The effects of mycotoxin ingestion are mainly chronic, implying hidden disorders with reduced ingestion, productivity and fertility [3,8,22]. Such effects cause severe economic losses through clinically ambiguous changes in animal growth, feed intake reduction or feed refusal, alteration in nutrient absorption and metabolism, effects on the endocrine system as well as suppression of the immune system [2,3,23,24,25].

Ruminants are less susceptible to mycotoxins than monogastrics, because of the rumen microbiota and the feed particles contained in the rumen compartment may be effective in the degradation, deactivation and binding of these toxic molecules, hence protecting animals [3,5,26,27,28,29,30].

**Table 1:** Number of Scopus database citations for several secondary metabolites produced by mycotoxigenic fungi and their scientific interests.

Secondary Metabolites	Scopus Citation	Scientific Interest ^a^	Secondary Metabolites	Scopus Citation	Scientific Interest ^a^
AAL toxin	100	**	Infectopyrones	3	*
Aflatoxins	16,939	*****	Islanditoxin	10	*
Aflavinine	12	*	Luteoskyrin	135	**
Agroclavine	214	***	Marcfortine A, B and C	38	*
Alternariol	396	****	Monacolins	242	***
Andrastins	30	*	Moniliformin	399	****
Aspergillic acid	66	*	Monoacetoxyscirpenol	64	*
Aurofusarin	55	*	Mycophenolic acid	241	***
Beauvericin	441	****	Neosolaniol	242	***
β-nitropropionic acids	4	*	Nivalenol	1014	*****
Botryodiploidin	36	*	Novae-zelandins	1	*
Butenolide	1337	*****	Ochratoxins	5162	*****
Byssochlamic acid	31	*	Oosporein	45	*
Chlamydosporol	21	*	Orsellinic acid	205	***
Chrysogine	18	*	Paspalitrems	7	*
Citreoviridin	124	**	Patulin	1606	*****
Citrinin	1994	*****	Penicillic acid	437	****
Citreoisocoumarin	9	*	Penitrem	202	***
Clavine alkaloids	146	**	Phomopsin	123	**
Culmorin	33	*	PR toxin	320	****
Cyclopiazonic Acid	2307	*****	PR-amide	6	*
Deoxynivalenol	3720	*****	PR-imine	5	*
Diacetoxyscirpenol	759	****	Pseurotins	56	*
Dicoumarol	3811	*****	Roquefortines	213	***
Diketopioperazines	1	*	Roridins	32	*
Eremofortin C	10	*	Rubratoxin	191	**
Ergot toxins	7567	*****	Rubrofusarin	75	*
Ergotamine	7298	*****	Scirpentriol	69	*
Festuclavine	74	*	Slaframine	103	**
Fumagillin	939	****	Sphingofungin	47	*
Fumigatins	23	*	Sporidesmin	207	***
Fumiquinazolines	56	*	Stachbotryotoxins	1	*
Fumitremorgen	11	*	Sterigmatocystin	1000	****
Fumitremorgines	357	****	T-2 & HT-2 toxin	470	****
Fumonisins	3542	*****	Tentoxin	208	***
Fusarenone-X	54	*	Tenuazonic acid	256	***
Fusaric Acid	675	****	Tremorgens	37	*
Fusarins	100	**	Tremorgens	46	*
Fusariocin	2	*	Trypacidin	20	*
Gliotoxin	996	****	Verruculogen	112	**
Helvolic acid	89	**	Zearalenone	3443	*****

^a^: The scientific interest associated to each secondary metabolite was assigned on the basis of number of Scopus citations obtained by using “Article title, Abstract, Keywords” document search criterion; *: for 1–99 citations; **: for 100–199 citations; ***: 200–299 citations; ****: 300–999 citations; *****: >1000 citations.

**Figure 1:**
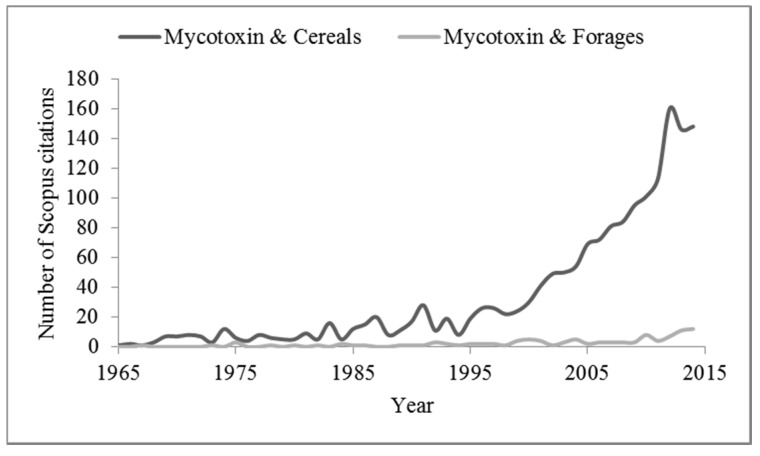
Number of Scopus database citations obtained by searching the keywords “Mycotoxins & Cereals” or “Mycotoxins & Forages”.

A summary of main toxic products from rumen metabolism and entity of reduction of mycotoxin biological potency were provided by Fink-Gremmels [31]. However, ruminant diets include starch (mainly cereals) and protein feeds, their by-products as well as grazed forage, hay or grass (GS), grass/legume (GLS), whole-crop forage maize (MS), small grain (SMS) and sorghum (SS) silages [32,33], which increase the risk of mycotoxin exposure compared to swine and poultry that have less varied diets. Some recent evidence suggests the greatest exposure to some regulated mycotoxins in cows could be related to forage contamination [10,34,35,36,37,38], even if this aspect remains poorly investigated. In particular, published articles where researchers investigated the presence of mycotoxins in hay and silages are very limited when compared to those analyzing the problem of mycotoxin contaminations in cereals (Figure 1). Furthermore, many other secondary metabolites different from regulated mycotoxins could be detected in forages, even if knowledge of their occurrence in forages is currently limited [10,28,37,39,40].

This review includes the knowledge of mycotoxins in cow feeds obtained in the last 15 years, with special emphasis on mycotoxins detected in forages, and animal toxicological issues due to their ingestion. In addition, the main strategies for preventing the problem of mycotoxin presence under farm conditions are presented and discussed.

## 2. Mycotoxin Occurrence in Animal Feeds, with Special Emphasis on Their Presence in Forages

From the 1970s, several reviews have been published in which occurrence data as well contamination levels of some mycotoxins in cereals and cereal by-products for animal nutrition have been reported [7,12,41,42,43,44,45,46,47,48,49,50,51] and nowadays more than 100 Countries have issued specific regulated or recommended limits or detailed guidelines for mycotoxin control in products intended for animal feeds [52,53,54,55,56]. In the last 15 years, an emerging issue related to mycotoxin contaminations of forages and factors affecting their occurrence at pre-harvest in the field or during ensiling and storage of forage crops has progressed. These aspects have been the basis of different review papers recently published [5,8,28,37,39,57,58,59,60].

Filamentous fungi can grow on forages and their presence is frequently observed in silage or hay [8,24,61,62]. Usually, the three most important toxigenic genera occurring pre-harvest are *Aspergillus*, *Fusarium* and perhaps *Alternaria* spp. [8]. In particular, the latter two are often categorized as field fungi whereas some species of *Aspergillus* can occur both pre- and post-harvest. The occurrence of these fungi in the field is related to several factors, including agricultural practices and climatic conditions [63,64]. During ensiling, most fungi can be eliminated [65,66]. However, there are other species, such as *Aspergillus fumigatus*, *Penicillium* *roqueforti*, *P. paneum*, *F. oxysporum* and *Monascus ruber* that are able to tolerate both high levels of organic acids and carbon dioxide in addition to low availability of oxygen [8,28,37,67,68]. In particular, presence of oxygen in some parts of silage during storage or oxygen penetration during feed-out and aerobic spoilage phases could allow mold growth and mycotoxin production. In high quality silage, lactic acid bacteria are effective in hindering any mold growth, but just a small raise in the oxygen concentration could provide the right growth conditions for fungi such as *P. roqueforti* and *P. paneum*. Indeed, if most of acetic and lactic acids as well as carbon dioxide evaporate and more oxygen is present, nearly all cereal-associated filamentous fungi may grow [8,69]. Considerable variability in the mycotoxin occurrences and concentration levels has been reported in forages and this could be probably due to a multitude of environment-related (*i.e.*, meteorological conditions, agronomical practices, ensiling procedures, management of forage, types of forage, *etc.*) or lab-related (sampling procedures, storage and preparation of samples, adopted analytical methods, *etc.*) factors. Results about occurrence and concentration levels of main mycotoxins detected in hay and silages are presented in Table 2. In Table 3, we also report mycotoxins analyzed but not detected in forages, to improve occurrence data discussion.

### 2.1. Alternaria Toxins in Forages

Different *Alternaria* species, such as *A. alternata*, *A. arborescens* and *A. tenuissima*, have been isolated from hay and silages [8,65]. However, Andersen *et al.* [70] recently suggested that *A. alternata* is a rare species and most strains originally identified as such in reality belong to *A. tenuissima* species-group, *A. arborescens* species-group or other *Alternaria* species-groups. These fungi produce a wide range of compounds, such as alternariols, altertoxins, altenuene, tentoxin and tenuazonic acid, with suspected but still unconfirmed toxic properties [71,72]. However, *A.* *infectoria* produces several other secondary metabolites, such as 4Z-infectopyrone, phomapyrones, novae-zelandins, dehydrocurvularin, pyrenochaetic acid or alternarienonic acid [70,73].

Only few reports on the natural occurrence of these compounds in feeds have been reported [74]. Among these, Yu *et al.* [75] reported high incidence of AAL type A toxins in different feeds, such as hay, hay silages and MS, with concentrations sometimes exciding 1000 μg/kg. These authors analyzed these mycotoxins by using an unspecific screening method, consisting in a direct competitive enzyme-linked immunosorbent assays, and as representative of mycotoxins produced by *A. alternata*. However, Andersen *et al.* [70] verified that only one strain of *A. arborescens* was associated with the production of AAL toxins and none of the other 98 strains of identified *A. arborescens* or other *Alternaria* species-groups produced these toxins. Storm *et al.* [24] reported low occurrences and low concentrations of alternariol and alternariol momomethyl in forages sampled in Denmark. No occurrence data were reported for other *Alternaria* secondary metabolites.

**Table 2:** Survey of mycotoxins detected in forages and other fibrous feeds from the literature.

Forage Products	Mycotoxins ^a^	Number of Samples	Incidence (%)	Mean (Excluding not Detectable Data when Possible)	Range or Maximal Detected Value	Nation	References	Notes
***Alternaria* spp. derived toxin**								
Different feeds	AAL TA toxin	63	97%	560 μg/kg	90–1470 μg/kg	WI, US	[75]	
MS	AAL TA toxin	60	~30%	170 μg/kg	200–2010 μg/kg	PA, US	[76]	
Hay and hay silage	AAL TA toxin	25	100%	720 μg/kg	290–1160 μg/kg	WI, US	[75]	
MS	AAL TB toxin	60	~15%	50 μg/kg	30–900 μg/kg	PA, US	[76]	
MS	Alternariol	82	2%	18 μg/kg	max 24 μg/kg	Denmark	[24]	
MS	Alternariol ME	82	2%	8 μg/kg	max 8.8 μg/kg	Denmark	[24]	
***Aspergillus flavus* and *A. parasiticus* derived toxin**								
MS	AFB_1_	1	-	28 μg/kg		France	[77]	
MS	AFB_1_	100	92%	-	0.6– > 4 μg/kg	Italy	[78]	only core samples
MS	AFB_1_	116	13%	33 μg/kg	2–54 μg/kg	Brazil	[62]	core samples
MS	AFB_1_	9	-	-	4–34 μg/kg	France	[79]	from 1 farm
Silages	β-nitropropionic acid	3	33%	1360 μg/kg	-	Netherlands	[16]	
**Various *Aspergillus* and *Penicillium* spp. derived toxin**								
Different feeds	Cyclopiazonic acid	63	87%	340 μg/kg	120–1820 μg/kg	WI, US	[75]	
Hay and hay silage	Cyclopiazonic acid	25	80%	390 μg/kg	120–1820 μg/kg	WI, US	[75]	
MS	Cyclopiazonic acid	120	37%	120 μg/kg	20–1430 μg/kg	PA, US	[80]	4 samples from 30 bunkers
Silages	Cyclopiazonic acid	3	33%	55 μg/kg	-	Netherlands	[16]	
***Aspergillus fumigatus* derived toxin**								
MS	Gliotoxin	1	-	4 μg/kg		France	[77]	
MS	Gliotoxin	90	-	5130 μg/kg	5100–6500 μg/kg	Argentina	[81]	
Silages	Gliotoxin	3	33%	1870 μg/kg	-	Netherlands	[16]	
MS	Gliotoxin	196	<1%	140 μg/kg	max 600 μg/kg	Italy	[69]	3 samples from 68 silos
***Fusarium* spp. derived toxin Trichothecenes type A**								
MS	15-acetyl DON	140	<1%	901 μg/kg	max 1013 μg/kg	Netherlands	[82]	over three years
MS	15-acetyl DON	5	100%	59 μg/kg	max 127 μg/kg	Germany	[83]	
MS	3-acetyl DON	20	0%	-	-	Denmark	[84]	
Hays	3-acetyl DON	28	4%	20 μg/kg	-	Germany	[83]	
MS	3-&5-acetyl DON	19	21%	217 μg/kg	135–300 μg/kg	Switzerland	[85]	
Different feeds	DON	63	100%	730 μg/kg	340–6020 μg/kg	WI, US	[75]	
Compound feed	DON	72	54%	433 μg/kg	max 2408 μg/kg	Netherlands	[36]	
MS	DON	20	100%	1056 μg/kg	160–5094 μg/kg	Denmark	[84]	
MS	DON	140	72%	854 μg/kg	max 3142 μg/kg	Netherlands	[82]	over three years
MS	DON	82	6%	1629 μg/kg	max 2974 μg/kg	Denmark	[24]	Quantitative analysis
MS	DON	1	-	146 μg/kg	-	France	[77]	
MS	DON	196	8%	280 μg/kg	max 560 μg/kg	Italy	[69]	3 samples from 68 silos
MS	DON	9	-	-	100–213 μg/kg	France	[79]	from 1 farm
MS	DON	5	100%	2919 μg/kg	max 3944 μg/kg	Germany	[83]	
MS	DON	19	100%	1356 μg/kg	780–2990 μg/kg	Switzerland	[85]	
MS	DON	116	24%	1610 μg/kg	150–3420 μg/kg	Brazil	[62]	core samples
Silages	DON	3	100%	396 μg/kg	max 761 μg/kg	Netherlands	[16]	
Ensiled by-products	DON	29	0%	-	-	Netherlands	[36]	
Feed commodities	DON	8	38%	1019 μg/kg	max 1811 μg/kg	Netherlands	[36]	
Forage products	DON	13	15%	348 μg/kg	max 489 μg/kg	Netherlands	[36]	
Hay and hay silage	DON	25	100%	610 μg/kg	510–720 μg/kg	WI, US	[75]	
Hays	DON	28	14%	41 μg/kg	max 69 μg/kg	Germany	[83]	
Silage	DON	47	53%	550 μg/kg	max 1250 μg/kg	Netherlands	[36]	
SGS (Wheat)	DON	30	10%	621 μg/kg	max 1165 μg/kg	Netherlands	[36]	over two years
MS	DON 2000	196	59%	1290 μg/kg DM	240–12,890 μg/kg DM	Germany	[86]	ELISA method
MS	DON 2002	182	89%	2100 μg/kg DM	260–14,290 μg/kg DM	Germany	[86]	ELISA method
MS	DON 2001	32	86%	800 μg/kg	max 3700 μg/kg	PA, US	[87]	over two years
MS	DON 2002	39	66%	1100 μg/kg	max 5100 μg/kg	PA, US	[87]	over two years
MS	Fusarenon X	20	20%	4 μg/kg	8–14 μg/kg	Denmark	[84]	
MS	Nivalenol	5	100%	1612 μg/kg	max 2809 μg/kg	Germany	[83]	
MS	Nivalenol	19	42%	521 μg/kg	190–760 μg/kg	Switzerland	[85]	
Hays	Nivalenol	28	4%	131 μg/kg	max 222 μg/kg	Germany	[83]	
MS	Nivalenol	82	13%	266 μg/kg	max 758 μg/kg	Denmark	[24]	Quantitative analysis
***Fusarium* spp. derived toxin: Trichothecenes type B**								
MS	15monoacetoxyscirpenol	5	60%	30 μg/kg	max 49 μg/kg	Germany	[83]	
MS	HT-2 toxin	20	60%	104 μg/kg	2–327 μg/kg	Denmark	[84]	
MS	HT-2 toxin	5	100%	18 μg/kg	max 26 μg/kg	Germany	[83]	
MS	HT-2 toxin	19	26%	95 μg/kg	76–120 μg/kg	Switzerland	[85]	
MS	T-2 toxin	20	5%	2 μg/kg	-	Denmark	[84]	
MS	T-2 toxin	19	42%	36 μg/kg	14–84 μg/kg	Switzerland	[85]	
***Fusarium* spp. derived toxin: Fumonisins**								
Different feeds	FB_1_	63	37%	280 μg/kg	20–2120 μg/kg	WI, US	[75]	
MS	FB_1_	140	1%	17,000 μg/kg	max 26,200 μg/kg	Netherlands	[82]	over three years
MS	FB_1_	86	97%	615 μg/kg	21–1824 μg/kg	IL, US	[88]	
MS	FB_1_	60	~75%	2020 μg/kg	200–10,100 μg/kg	PA, US	[76]	
MS	FB_1_	116	15%	5440 μg/kg	300–3400 μg/kg	Brazil	[62]	core samples
MS	FB_1_	100	88%	-	900– > 10,000 μg/kg	Italy	[78]	only core samples
Hay and hay silage	FB_1_	25	52%	120 μg/kg	20–450 μg/kg	WI, US	[75]	
Silages	FB_1_	3	33%	21 μg/kg	-	Netherlands	[16]	
MS	FB_2_	64	72%	93 μg/kg	21–276 μg/kg	IL, US	[88]	
MS	FB_2_	60	~40%	980 μg/kg	200–20,300 μg/kg	PA, US	[76]	
MS	FB_3_	51	57%	51 μg/kg	16–161 μg/kg	IL, US	[88]	
***Fusarium* spp. derived toxin: other *Fusarium* toxins**								
GS (bunkers)	Beauvericin	88	-	~30 μg/kg DM	-	Ireland	[89]	
GS (round bale)	Beauvericin	56	-	~30 μg/kg DM	-	Ireland	[89]	
MS	Enniatin A1	6	-	~120 μg/kg DM	-	Ireland	[89]	
GS (bunkers)	Enniatin A1	88	-	~40 μg/kg DM	-	Ireland	[89]	
GS (bunkers)	Enniatin A1	88	-	~20 μg/kg DM	-	Ireland	[89]	
GS (round bale)	Enniatin A1	56	-	~25 μg/kg DM	-	Ireland	[89]	
MS	Enniatin B	82	24%	53 μg/kg	max 152 μg/kg	Denmark	[24]	
GS (bunkers)	Enniatin B	88	-	~60 μg/kg DM	-	Ireland	[89]	
GS (round bale)	Enniatin B	56	-	~250 μg/kg DM	-	Ireland	[89]	
MS	Enniatin B1	6	-	~160 μg/kg DM	-	Ireland	[89]	
GS (bunkers)	Enniatin B1	88	-	~180 μg/kg DM	-	Ireland	[89]	
GS (round bale)	Enniatin B1	56	-	~80 μg/kg DM	-	Ireland	[89]	
***Fusarium* spp. derived toxin: Zearalenone**								
MS	α-ZOL	5	20%	15 μg/kg	-	Germany	[83]	
MS	β-ZOL	5	20%	116 μg/kg	-	Germany	[83]	
Different feeds	ZEA	63	32%	220 μg/kg	120–310 μg/kg	WI, US	[75]	
Compound feed	ZEA		28%	80 μg/kg	max 363 μg/kg	Netherlands	[36]	
MS	ZEA	140	49%	174 μg/kg	max 943 μg/kg	Netherlands	[82]	over three years
MS	ZEA	82	28%	66 μg/kg	max 311 μg/kg	Denmark	[24]	Quantitative analysis
MS	ZEA	9	-	-	23–41 μg/kg	France	[79]	from 1 farm
MS	ZEA	5	100%	432 μg/kg	max 1790 μg/kg	Germany	[83]	
MS	ZEA	19	79%	180 μg/kg	83–430 μg/kg	Switzerland	[85]	
MS	ZEA	85	15%	-	>50 μg/kg	Italy	[90]	
MS	ZEA	100	60%	-	30–>300 μg/kg	Italy	[78]	only core samples
Silages	ZEA	3	100%	145 μg/kg	max 240 μg/kg	Netherlands	[16]	
Ensiled by-products	ZEA		-	-	-	Netherlands	[36]	
Feed commodities	ZEA		38%	80 μg/kg	max 108 μg/kg	Netherlands	[36]	
Forage products	ZEA		8%	82 μg/kg	-	Netherlands	[36]	
GS	ZEA	120	6%	936 μg/kg	max 308 μg/kg	Netherlands	[82]	over three years
Hay and hay silage	ZEA	25	0%	-	-	WI, US	[75]	
Hays	ZEA	28	43%	24 μg/kg	max 115 μg/kg	Germany	[83]	
Hays	ZEA	44	21%	-	-	Ireland	[91]	
Haylages	ZEA	40	8%	-	-	Ireland	[91]	
Hays	ZEA	65	8%	-	-	Canada	[91]	
Silage	ZEA		17%	125 μg/kg	max 273 μg/kg	Netherlands	[36]	
***Penicillium* spp. derived toxin**								
Different feeds	PR toxin	63	76%	130 μg/kg	50–260 μg/kg	WI, US	[75]	
Hay and hay silage	PR toxin	25	80%	15 μg/kg	50–260 μg/kg	WI, US	[75]	
GS (round bale)	16-OH-roquefortine C	5	20%	-	range 100–1000 μg/kg	Ireland	[10]	
MS	Andrastin A	82	18%	169 μg/kg	max 691 μg/kg	Denmark	[24]	Quantitative analysis
GS (round bale)	Andrastin A	56	-	~500 μg/kg DM	-	Ireland	[89]	
GS (round bale)	Andrastin A	5	100%	-	range trace-20,000 μg/kg	Ireland	[10]	
MS	Citreoisocoumarin	82	8%	-	-	Denmark	[24]	Qualitative analysis
GS (round bale)	Citreoisocuomarin	5	40%	-	trace	Ireland	[10]	
MS	Marcfortine A	82	7%	-	-	Denmark	[24]	Qualitative analysis
GS (round bale)	Marcfortine A	5	60%	-	range 100–1000 μg/kg	Ireland	[10]	
MS	Marcfortine B	82	1%			Denmark	[24]	Qualitative analysis
GS (round bale)	Agroclavine	5	40%	-	range 100–1000 μg/kg	Ireland	[10]	from *A. fumigatus* too [92]
GS (round bale)	Festuclavine	5	40%	-	range 100–1000 μg/kg	Ireland	[10]	from *A. fumigatus* too [92]
MS	Mycophenolic Acid	135	28%	690 μg/kg	20–23,000 μg/kg	Germany	[93]	
MS	Mycophenolic acid	120	42%	160 μg/kg	20–1300 μg/kg	PA, US	[80]	4 samples from 30 bunkers
MS	Mycophenolic acid	82	2%	8 μg/kg	max 8.8 μg/kg	Denmark	[24]	Quantitative analysis
MS	Mycophenolic Acid	196	8%	1760 μg/kg	max 48,000 μg/kg	Italy	[69]	Three samples from 68 silos
Silages	Mycophenolic Acid	3	100%	4244 μg/kg	max 7565 μg/kg	Netherlands	[16]	
Ensiled by-products	Mycophenolic acid		10%	66 μg/kg	max 83 μg/kg	Netherlands	[36]	
GS (bunkers)	Mycophenolic Acid	88	-	~250 μg/kg DM	-	Ireland	[89]	
GS (round bale)	Mycophenolic Acid	56	-	~1250 μg/kg DM	-	Ireland	[89]	
GS	Mycophenolic Acid	98	37%	2200 μg/kg	20–35,000 μg/kg	Germany	[93]	
GS (round bale)	Mycophenolic acid	5	100%	-	range trace-20,000 μg/kg	Ireland	[10]	
Silage	Mycophenolic acid		13%	524 μg/kg	max 2630 μg/kg	Netherlands	[36]	
MS	Roquefortine C	12	8%	200 μg/kg DM	-	Germany	[94]	molded silages
MS	Roquefortine C	12	100%	17,000 μg/kg DM	700–36,000 μg/kg DM	Germany	[94]	unmolded samples
MS	Roquefortine C	60	30%	5470 μg/kg	50–28,000 μg/kg DM	Germany	[95]	data of Armbruster, 1994
MS	Roquefortine C	120	60%	380 μg/kg	10–5710 μg/kg	PA, US	[80]	4 samples from 30 bunkers
MS	Roquefortine C	82	2%	173 μg/kg	max 189 μg/kg	Denmark	[24]	Quantitative analysis
MS	Roquefortine C	196	5%	740 μg/kg	max 32,000 μg/kg	Italy	[69]	3 samples from 68 silos
Ensiled by-products	Roquefortine C		7%	123 μg/kg	max 170 μg/kg	Netherlands	[36]	
GS (bunkers)	Roquefortine C	88	-	~500 μg/kg DM	-	Ireland	[89]	
GS (round bale)	Roquefortine C	56	-	~280 μg/kg DM	-	Ireland	[89]	
GS	Roquefortine C	24	13%	-	range 10–580 μg/kg	Germany	[10]	From Ambruster, 2008 PhD thesis
GS	Roquefortine C	20	15%	280 μg/kg	range 10–580 μg/kg	Germany	[95]	From Ambruster, 2008 PhD thesis
GS	Roquefortine C	120	<1%	81 μg/kg	-	Netherlands	[82]	over three years
GS (round bale)	Roquefortine C	5	40%	-	range 1000–20,000 μg/kg	Ireland	[10]	
Silage	Roquefortine C		19%	778 μg/kg	max 3160 μg/kg	Netherlands	[36]	
GS (wilted)	Roquefortine C	12	75%	200 μg/kg DM	100–300 μg/kg DM	Germany	[94]	molded silages
GS (wilted)	Roquefortine C	12	42%	600 μg/kg DM	200–15,000 μg/kg DM	Germany	[94]	unmolded samples
MS	Roquefortine A	82	11%	-	-	Denmark	[24]	Qualitative analysis
GS (round bale)	Roquefortine A	5	40%	-	range 100–1000 μg/kg	Ireland	[10]	
GS (round bale)	Roquefortine B	5	40%	-	range 100–1000 μg/kg	Ireland	[10]	
GS (round bale)	Roquefortine D	5	40%	-	range 100–1000 μg/kg	Ireland	[10]	
MS	Patulin	120	23%	80 μg/kg	10–1210 μg/kg	PA, US	[80]	4 samples from 30 bunkers
Silages	Patulin	3	100%	153 μg/kg	max 211 μg/kg	Netherlands	[16]	
***Monascusruber* derived toxin**								
Silages	Monacolin K_B_	233	21%	6161 μg/kg	28–65,400 μg/kg	Germany	[96]	
Silages	Monacolin K_L_	233	19%	1767 μg/kg	25–15,600 μg/kg	Germany	[96]	
MS	Citrinin	1	-	12 μg/kg		France	[77]	
MS	Citrinin	9			4–25 μg/kg	France	[79]	from 1 farm
Silages	Citrinin	233	6%	9 μg/kg	2–64 μg/kg	Germany	[96]	

^a^: AAL TA toxin, *Alternaria alternata* toxins type A; AAL TB toxin, *Alternaria alternata* toxins type A; aflatoxin B_1_, AFB_1_; Alternariol ME, alternariol monomethyl ether; deoxynivalenol, DON; fumonisin B_1_, FB_1_; fumonisin B_2_, FB_2_; fumonisin B_3_, FB_3_; grass silage, GS; whole-crop forage maize silage, MS; ochratoxin A, OTA; whole-crop small grain cereal silage, SGS; α-zearalenol, α-ZOL; β-Zearalenol, β-ZOL; zearalenone, ZEA.

**Table 3:** Survey of mycotoxins not detected in forages from the literature.

Forage Products	Mycotoxins ^a^ not Detected	References
MS	AFB_1_, AFB_2_, AFG_1_, AFG_2_, 3-acetyl-DON, DAS, ergotamin, FB_2_, fusarenon-X, OTA, mycophenolic acid, penicillic acid, roquefortin C, sterigmatocystin, T-2 toxin, HT-2 toxin	[82]
MS	AFB_1_, AFB_2_, AFG_1_, AFG_2_, OTA, T-2 toxin, HT-2 toxin, 3-acetyl-DON, 15-acetyl-DON, DAS, sterigmatocystin, fusarenon-X, ergotamine, penicillic acid	[36]
MS	Cyclopiazonic acid, fumitremorgin A, gliotoxin, OTA, patulin, penitrem A, sterigmatocystin, T-2 toxin, tenuazonic acid, altersetin, fumigaclavine A, fumigaclavine C, PR toxin	[24]
MS	ZEA, PR toxin	[69]
MS	3-acetyldeoxynivalenol, DAS, fusarenon-X, T-2 toxin, HT-2 toxin, neosolaniol, scirpentriol	[83]
Hays	15-monoacetoxyscirpenol, 15-acetyldeoxynivealenol, DAS, fusarenon-X, T-2 toxin, neosolaniol, scirpentriol, α-ZOL, β-ZOL	[83]
MS	FB_1_, OTA, ZEA	[77]
MS	Gliotoxin, OTA	[79]
Hays and haylages	FBs, AFs, T-2 toxin, OTA	[91]

^a^: aflatoxin B_1_, AFB_1_; aflatoxin B_2_, AFB_2_; aflatoxin G_1_, AFG_1_; aflatoxin G_2_, AFG_2_; deoxynivalenol, DON; diacetoxyscirpenol, DAS; fumonisin B_1_, FB_1_; fumonisin B_2_, FB_2_; fumonisins, FBs; whole-crop forage maize silage, MS; ochratoxin A, OTA; α-zearalenol, α-ZOL; β-Zearalenol, β-ZOL; zearalenone, ZEA.

### 2.2. Aspergillus Toxins in Forages

Presence of *Aspergillus flavus* and *A. parasiticus* has been reported in ensiled products, such as MS and high moisture maize, and the most important mycotoxins produced by these organisms are AFs (AFB_1_, AFB_2_, AFG_1_ and AFG_2_). Sporadically, these toxins were detected at low levels in forages thus contributing to increase AFB_1_ intake level in lactating dairy cows [77,78,79]. Otherwise, AFB_1_ was not quantified in silages such as MS, GS or SS [36,82,97]. However, AFs produced on growing crops may not be uniformly distributed across the field and when samples are collected, they could or could not be representative of the location in which the AFs are present and, consequently, of the AFs distribution in ensiled mass [28]. Therefore, reliability of measurements is strongly affected by protocols adopted to collect representative samples, to prepare samples for analysis or to extract and quantify mycotoxins [98,99,100]. Because of the heterogeneous distribution of AFs [101] and more generally of all mycotoxins [102], the variability associated with mycotoxin test procedures usually depends mainly by sampling plan. For these aspects, the European Commission set the methods of sampling and analysis for official control of the levels of mycotoxins in foodstuff [103] or in cereals, cereal products and compound feeds for animal feeding [53,104]. Nothing is currently done by authorities to set specific sampling procedures for hay or silages.

Additional mycotoxins produced by *A. flavus* and other *Aspergillus* species are kojic, cyclopiazonic and β-nitropropionic acids [105], but their presence is sporadically reported in silages [24]. In particular, Santos and Fink-Gremmels [16] reported β-nitropropionic acid in one of three sampled silages in Netherlands, at a concentration of 1360 μg/kg. However, no documentation on the analytical detection of this compound was provided.

*Aspergillus fumigatus* is one of the main mycotoxigenic fungus infecting forages under warm conditions [8,92,106]. Risk of presence of its related toxins has been reported particularly in silages and it is capable of producing more than 226 potentially bioactive secondary metabolites [92]. Among these, gliotoxin is clearly the most toxic metabolite and it has most often been analyzed to indicate presence of *A. fumigatus* toxin metabolites in silages [69,77,81]. However, it is mainly believed to be produced during infections of mammalians [107]. Storm *et al.* [8] reported that gliotoxin is mainly produced on substrates characterized by a low C to N ratio, therefore it does not represent a good marker of *A. fumigatus* presence. The low incidence of gliotoxin reported for MS sampled in Italy could be presumably related to this aspect [69]. Unfortunately, most other compounds from this fungus have not been assayed in silages and we are unable to report occurrence data. Storm *et al.* [24] recently discussed the absence of several *A. fumigatus* derived mycotoxins, such as gliotoxin, fumitremorgin A, fumigaclavines A and C in MS sampled in Denmark. Boundra and Morgavi [108] reported that gliotoxin, helvolic acid and verruculogen are stable during forage storage, whereas fumagillin was unstable under ensiling conditions. Agroclavine and festuclavine are other mycotoxins potentially produced by *A. fumigatus* [92].

Cyclopiazonic acid is a toxic indole tetramic acid, first isolated from *Penicillium griseofulvum* and subsequently from other *Penicillium* species, *A. flavus* and *A. oryzae*. Because this toxin can be produced by *A. flavus* too, co-occurrence with AFs and β-nitropropionic acid has been suspected. Only limited studies were published on cyclopiazonic acid occurrence in forages and it was detected in 37% of MS [80] and 80% of hays and hay silages [75] sampled in US, with contaminations exceeding 1000 μg/kg.

Several fungi of the genera *Aspergillus* and *Penicillium* spp. can produce OTA, including *A. westerdijkiae*, *A. niger*, *A. fresenii*, *A. carbonarius*, *P. verrucosum* and *P. nordicum* [109,110,111,112]. Many authors did not detect OTA in forages [24,67,82,91], first at all because these fungi do not tolerate high concentrations of acetic acid and CO_2_ [109,110,113]. Lastly, maltoryzine is produced by *A. clavatus.* However, data of its occurrence in forages are not available.

### 2.3. Fusarium Toxins in Forages

Among *Fusarium* derived mycotoxins, trichothecenes type A and B are produced by several species [24,85,114]. Among trichothecenes type B, the most studied mycotoxins are DON and to less extent nivalenol and fusarenon-X as well as their acetylated and deacetylated analogues (3-acetyl-DON, 15-acetyl-DON and others). They are primarily produced by *F. culmorum* and *F. graminearum* [115]. DON is considered the most prevailing mycotoxin in silages and other forages [8,116] and it can be present at different incidence rates and at different concentration levels. In particular, incidences of DON in forages higher than 80% were reported in North America [75,117] and North Europe [24,36,83,84,86] with average contamination levels highly variable, but in some cases exceeding 2000 μg/kg [83,86]. The distribution of DON could differ in silos, even if this aspect has not been yet clarified [28]. For instance, Richard *et al.* [15] measured higher DON concentrations in upper than bottom parts of silos, whereas the same authors reported opposite data two years later [77]. Furthermore, other authors [36,69] did not describe any sampling zone effects for neither DON nor ZEA. The incidences of nivalenol could range from 100% [86] to 13% [24] of collected MS, whereas Schollenberger *et al.* [83] reported an incidence of 4% with average nivalenol concentration level of 131 μg/kg in hays. Lastly, Storm *et al.* [24] reported about 20% of collected MS were contaminated by fusarenon X at a level lower than 5 μg/kg.

Mycotoxins such as diacetoxyscirpenol (DAS), T-2 and HT-2 toxins and their de-acetylated analogues belong to type A trichothecenes and they are mainly produced by *F. poae*, *F. sporotrichioides* and *F. langsethiae* [115]. Despite some authors [24,83,85] reported these trichothecenes were often detected in MS, the average concentration levels should be normally considered very low. T-2 toxin was not detected in hay and MS collected in Germany, as well as DAS and its acetylated compounds [83].

FBs are primarily produced by *F. proliferatum* and *F. verticillioides* [115] and their contamination in pre-harvest crops is often reported [8,118]. Among FBs, FB_1_ is the predominant and most studied one. For FB_1_, incidences higher than 30% were reported in MS sampled in North America [75,76,119], whereas in the Netherlands and France the occurrence was low [15,82]. About 50% of hay and hay silages sampled in Wisconsin were contaminated by FB_1_ [75], with average concentration of 120 μg FB_1_/kg. Other FBs, such as FB_2_ and FB_3_ were also detected in MS, but at very low contamination levels [65,119].

Several authors reported ZEA incidence data in MS, GS, hay or other feeds. On average, the 52% of collected MS resulted contaminated by ZEA with average contamination levels lower than 500 μg/kg [24,78,82,83,85]. ZEA was detected in the 43% of hay collected in Germany [83]. Contrarily, Richard *et al.* [77] and Gallo *et al.* [69,120] did not detect ZEA in MS. Other *Fusarium* derived toxins, such as beauvericins, enniatins and moniliformin were detected in silages (GS round bale, GS in bunkers or MS) both in Ireland and Denmark, but at very low contamination levels [24,89,121]. A degradation process occurring during ensiling was suspected but not still proved [8].

Concerning stability of *Fusarium* toxins, Boudra and Morgavi [122] reported that the concentrations of DON, FBs and ZEA decreased during ensiling in MS. Depending on DM content of silages, length of ensiling and temperature, toxin disappearances could range from 50% for ZEA to 100% for DON [28]. Furthermore, plants are able to modified mycotoxins by conjugation to polar substances [123,124,125,126]. Different *Fusarium* toxins, such as ZEA, nivalenol, T-2 and HT-2 toxins or FBs could contaminate feeds in their modified forms [118]. However, no data on presence of modified mycotoxins were reported for forages [126,127].

### 2.4. Penicillium Toxins in Forages

Species belonging to *Penicillium* section *Roquefortorum* such as *P. roqueforti* and *P. paneum* [11,110] are considered some of the most prevailing post-harvest fungi found in silages [15,37,81,84,128]. Different critical factors, such as unfavorable weather or storage conditions, could promote fungal growth and mycotoxin production [15,108]. A list of main mycotoxins produced by *Penicillium* strains was reported by Nielsen *et al.* [11]. Surely, these mycotoxins are the most researched and detected in forages. As reported by Auerbach *et al.* [94], *P. roqueforti* was isolated from 89% of visibly-moldy and from 85% of visibly-unmoldy silages. Similarly, *P. roqueforti* and *P. paneum* were isolated from 96% of MS stored in bunker silos or as silage stacks laid on soil [84]. In a survey conducted in the Netherlands [36], incidences of mycotoxins produced by *P. roqueforti* were reported both in MS and GS, being respectively 50% and 19% of collected samples. PR toxin, a mycotoxin produced by *P. roqueforti* [11], was detected in several feeds collected in North America. In particular, Yu *et al.* [75] reported an incidence of 76% of PR toxin in 63 feed samples (*i.e.*, 25 hays and 38 silages and mixed feeds), with an average contamination of 130 μg/kg. However, the employed methodology was a low specific immunochemical screening method and successive studies have not been able to verify the detected levels of this mycotoxin. A survey was carried out in Italy where 68 MS were sampled and authors did not detect presence of PR toxin [69].

Mycophenolic acid and roquefortines could be considered the most studied *Penicillium* derived compounds in ensiled products. The first, produced by *P. roqueforti* and *B. nivae* [8], was detected in MS with variable incidences, being about 40% [80], 30% [93], 10% [69] or lower than 3% [24,84]. Furthermore, mycophenolic acid concentrations higher than 20,000 μg/kg were reported [69,93,94]. Recently, Santos and Fink-Gremmels [16], sampling three GS in different herds in the Netherlands with visible aerobic instability and mold visible in all parts of silo bunkers, detected mycophenolic acid in all samples at levels ranging from 588 to 7565 μg/kg. Among roquefortines, produced by different strains of *P.* section *Roquefortorum*, the most studied was roquefortine C and this toxin was detected in more than 40% of sampled silages [10,80,94]. Otherwise, low incidence of roquefortine C was reported in MS [24] and GS [82]. High concentration values (> 20,000 μg/kg) were sporadically reported [69,94]. In particular, Driehuis *et al.* [36] reported average contaminations of 778 μg/kg for roquefortine C and 524 μg/kg for mycophenolic acid in silages, with maximum levels up to 3160 and 2630 μg/kg, respectively. Other roquefortines exclusively produced by *P. roqueforti* [11] were detected in 2 of 5 analyzed GS by O’Brien *et al.* [10] with average concentrations of 100–1000 μg/kg. For these mycotoxins, different authors [36,69] reported higher incidences as well as higher concentrations in MS collected from peripheral than core zones of silos.

Other *Penicillium* derived exometabolites have been detected in silages, such as andrastin A, citreoisocumarin, agroclavine, festuclavine and the *P. paneum* biomarker marcfortine A [10,24]. In particular, O’Brien *et al.* [10] reported 2 of 5 collected MS were contaminated by agroclavine and festuclavine produced by *Penicillium* strains, with concentrations ranging from 100 to 1000 μg/kg. Storm *et al.* [24] reported incidences of andrastin A, citreoisocumarin and marcfortine A lower than 20% in 82 collected MS from Denmark. Patulin, produced by *P. paneum* and *B. nivae* [8], was detected in 23% of MS with concentrations ranging from 10 to 1210 μg/kg. No information is currently available for other *Penicillium* derived toxin such as botryodiploidin [11].

### 2.5. Monascus Ruber Toxins in Forages

In *Monascus ruber* infected silage, citrinin has been detected [77,79,96]. Among other exometabolites produced by *M. ruber*, Schneweis *et al.* [96] reported that monacolin (statin family, cholesterol lowering) was detected in about 20% of collected silages.

### 2.6. Zygomycetes Fungi in Forages

Some Zygomycetes can, via endophytic bacteria, produce secondary metabolites and toxic rhizonins [9] and rhizonines, but these have not been found in silages. Jensen *et al.* [129] reported these fungi could cause zygomycosis in immunosuppressed animals.

## 3. Effect of Mycotoxins Ingestion on Ruminants: *In Vitro* and *in Vivo* Experiences

As introduced above, ruminants are considered to be less susceptible to negative effects of mycotoxins than monogastrics, rumen microflora and feed particles contained in rumen being effective in the degradation, deactivation or binding of these toxic molecules [3,29,130,131,132] and rumen microorganisms being able to reduce development of pathogens [133,134]. The mechanisms of action and toxic properties of several mycotoxins frequently detected in concentrates and forages have been studied. We refer to Table 11.1 of the CAST report [4] and the review of Hussein [135] for details. Furthermore, the cytotoxicity of several mycotoxins detectable in forages and produced by *Aspergillus* *fumigatus*, *Alternaria tenuissima*, *F. avenaceum*, *F. graminarum*, *P. roqueforti*, *P. paneum*, *M. ruber* or *B. nivea*, as well as cytotoxic effects of fungal agar or silage extracts were tested *in vitro* by Rasmussen *et al.* [136].

Actually, there are limited scientific evidences regarding the negative effects of mycotoxin ingestion on the health status and performance of cattle and the evaluation of the real economic impact of mycotoxins on ruminant livestock production system still represents a main issue that deserves further investigation [2,3,137,138,139,140]. We present in Table 4 the majority of *in vitro* published works where researchers investigated the effects of mycotoxin presence on rumen microbiota, whereas in Table 5 are summarized results from several *in vivo* studies designed to investigate effect of mycotoxin ingestion in ruminants.

**Table 4:** Survey on the effects of mycotoxins on rumen microbiota tested by *in vitro* approaches from literatures.

Mycotoxins ^a^	Media	Tested Dosages	Effects	References
AFB_1_	rumen fluid	0, 300, 600, 900 ng AFB_1_/mL buffered rumen fluid	↓ gas production, ↓ dry matter digestibility, ↓ NH_3_-N concentrations	[141]
AFB_1_	rumen fluid	1, 10 μg AFB_1_/mL buffered rumen fluid	↓ dry matter digestibility	[142]
AFB_1_	rumen fluid	9.5 ng AFB_1_/mL buffered rumen fluid	no effects	[143]
AFB_1_	rumen fluid	0, 320, 640, 960 ng AFB_1_/mL buffered rumen fluid	↓ final gas production, ↓ rate of degradation, ↓ NH_3_-N concentrations, ↑ isobutyrate, valerate and isovalerate molar proportions	[144]
DON	rumen fluid	0.36/0.46 or 5.76/6.90 mg of DON/kg diet	None, expect ↓ NDF digestibility	[145]
DON	rumen fluid	0.3 or 3.4/4.4 mg of DON/kg diet	None, expect ↓ NDF digestibility	[146]
DON	rumen fluid	40 μg DON/mL of rumen fluid	↓ gas production, ↓ VFA and NH_3_-N concentrations	[147]
DON and fusaric acid	culture media		antimicrobial activity of fusaric acid against *Ruminococcus albus* and *Methanobrevibacter ruminantium*. No effect of DON	[148]
Gliotoxin	rumen fluid	0, 1, 2, 5, 10, 20, 40, 80 μg/mL buffered rumen fluid	< 80 μg/mL no effects. At 80 μg/mL ↓ DM degradation, gas and VFA productions	[149]
FB_1_	rumen fluid	0, 50 or 100 mg/kg rumen fluid	none	[150]
OTA	rumen fluid	200 μg of OTA/l of rumen fluid	none	[151]
Patulin	rumen fluid	20, 100 and 300 μg of Patulin/mL rumen liquid	↓ Acetic acid production within 4 h and Inhibition of protein synthesis	[152]
Patulin	rumen fluid	0, 10, 20 and 40 mg of Patulin/mL rumen fluid	↓ dDM, VFA production, dNDF, dADF, dCHO, dCP and bacterial N flows ↑ NH3-N	[153]
Mycopenolic acid, Roquefortine C and PR toxin	rumen fluid	0.01, 0.30, 1.01, 1.71 and 2.00 μg of each mycotoxin/mL buffered rumen fluid	Mychopenolic acid and roquefortine C ↓ gas production, VFA production. No effect of PR toxin	[130]
Citrinin, Monacolin K, Pravastatin and Mevastatin	rumen fluid	5 or 20 μg of monacolin/mL rumen fluid; 5 or 20 μg of citrinin/mL rumen fluid; *Monascus* spp. contaminated rice	none, ↓Methane production	[154]

^a^: aflatoxin B_1_, AFB_1_; ammonia nitrogen, NH_3_-N; dADF, digestible ADF; dCHO; digestible carbohydrates; dDM, digestible dry matter; deoxynivalenol, DON; DM, dry matter; dNDF, digestible NDF; fumonisin B_1_, FB_1_; ochratoxin A, OTA; VFA, Volatile fatty acids.

**Table 5:** Survey on the effects of mycotoxins ingestion in ruminants from literatures (Field trial or FT and Experimental trial or ET).

Mycotoxins ^a^	Study	Animals	Tested Dosages	Reported Effects	References	Notes
AFB_1_	FT	Beef	0.2, 0.4, 0.6 or 0.8 mg of AFB_1_/kg of BW	↓ rumen mobility	[155]	
AFB_1_	FT	Beef	0, 100, 300, 700 and 1000 μg AFB_1_/kg diet	For levels 700 and 1000 μg/kg: Growth inhibition, ↓ feed efficiencies, ↑ liver and kidney weights	[156]	
AFB_1_	FT	Lactating dairy cows	20 μg AFB_1_/kg diet	↓ feed consumption, ↓ milk production	[157]	
AFB_1_	FT	Lactating dairy cows	120 μg AFB_1_/kg diet	↓ reproductive efficiency, ↓ milk production	[158]	
AFB_1_	FT	Lactating dairy cows	100 μg AFB_1_/kg diet	↓ milk production	[159]	
AFB_1_	ET	Lactating dairy cows	100 and 300 μg of AFB_1_/kg of BW	↓ feed intake → ↓ milk production	[160]	
AFB_1_	ET	Lactating dairy cows	13 mg of AFB1 (pure and impure from *Aspergillus parasiticus* in culture)	↓ milk production	[161]	
AFB_1_	ET	Sheep	1.8 and 2.4 mg of AFB_1_/kg diet	none	[162]	Exposition period of 5 years
AFB_1_	FT	Sheep	0.75 mg of AFB_1_/kg diet	Inappetence, apathy, hepatic lesion, neurological signs and death.	[163]	
AFB_1_	ET	Lambs	2.6 mg of AFB_1_/kg diet	↓ BW ↑ AST, GGT, prothrombin time, cholesterol, uric acid and triglyceride values ↓ albumin, glucose and urea nitrogen and urea-to-creatine ratio	[164]	
AFB_1_	ET	Lambs	2 mg of AFB_1_/kg diet	= BW ↓ ADG, immune response	[165]	
AFB_1_	ET	Lambs	350 μg AFB_1_/kg diet	= ADI and blood parameters ↓ ADG gain, serum Ca and P	[166]	Exposition period of 150 days
AFB_1_	ET	Lambs	0, 5.9, 11.8, 17.7, 23.5 μg AFB_1_/kg diet	= DMI, cellular immunity ↓ ADW	[167]	
AFB_1_	ET	Lambs	2.5 mg of AFB_1_/kg diet	↓ feed intakes, daily gain, and gain/feed ↑ AST, GGT, total protein, cholesterol	[168]	
AFB_1_	ET	Lactating dairy cows	96 μg/cow/day	slightly ↑ GGT and serum protein	[169]	
AFB1&FB_1_+FB2	ET	Heifers	C (1.9 μg of AFB_1_ and 3.8 mg of FBs/kg diet), A (12.0 μg of AFB_1_ and 6.6 mg of FBs/kg diet), A-F (19.9 μg of AFB_1_ and 23.2 mg of FBs/kg diet) diets	= BW, DMI ↑GGT delay in reproductive career	[170]	
AFB1, DON, ZEA, FB_1_, OTA, T-2 toxin	ET	Lactating dairy cows	38 AFB_1_ and 270 T-2 μg/kg; 720 DON, 701 FB_1_, 541 ZEA, 501 OTA mg/kg	↓ DMI, milk yield, CP and NDF digestibilities, impact on haematological parameters and immunosuppression	[171]	
Maltoryzine		Lactating dairy cows	unknown	general poison	[172]	
β-nitropropionic acid		Sheep and Cattle	unknown	emphysema and difficulty in locomotion	[173]	
DON	ET	Lactating and no lactating dairy cows	0.3 or 3.4/4.4 mg of DON/kg diet	↓ NDF digestibility and slightly ↓ in microbial crude protein	[146]	Two level of F:C ratio, being 40:60 or 70:30
DON	ET	Lactating dairy cows	4.4 or 5.3 mg DON/kg DM	↑ DMI ↓ Milk Fat	[174]	
DON	ET	Lactating dairy cows	4.4 or 5.3 mg DON/kg DM	↑ valerate ↓ pH, acetate and isobutyrate	[175]	
DON	ET	Lactating dairy cows	0.59, 42, and 104 mg of DON/cow/day	none	[176]	
DON	ET	Lactating dairy cows	8 mg of DON/kg diet	none	[177]	
DON	ET	Non lactating cows	about 8 or 35 mg of DON/cow/day	none, except slightly ↓ ingestion of contaminated feed	[178]	
DON	ET	Lactating dairy cows	66 mg of DON/kg diet	none	[176]	
DON	ET	Non lactating cows	4 or 3.6 mg of DON/kg diet and 0.13 or 0.05 mg of ZEA/kg in experiments 1 and 2, respectively	= rumen pH and VFA production ↓ microbial protein and ↑rumen NH3-N concentration and	[179]	
DON	ET	Lactating dairy cows	3.5 mg of DON/kg diet and 0.24 mg of ZEA/kg diet	= DMI and milk production; Influence on metabolic parameters and immune response	[180,181]	
DON	ET	Lactating dairy cows	Group CON (0.02 mg ZEA and 0.07 mg DON/ kg DM), group FUS-50 (0.33 mg ZEA and 2.62 mg DON/kg DM), group FUS-100 (0.66 mg ZEA and 5.24 mg DON/ kg DM)	none	[182]	
DON	ET	Lactating dairy cows	The average daily intake of DON in group K was 12.4 mg, in group T 14.1 mg and in group M 14.3 mg and ZEA in group K was 12.4 mg, in group T 0.67 mg and in group M 0.68 mg	slightly ↑ in AST and LDH	[183]	
AFB_1_ & DAS	ET	Lambs	Group control (uncontaminated), group AFB_1_-contaminated (2.5 mg /kg), group DAS-contaminated (5 mg/kg from chemical standard) and group AFB_1_/DAS co-contaminated (2.5 mg of AFB_1_ and 5 mg of diet/kg) diets	↓ Feed ingestion, BW	[184]	
FBs	ET	Lactating dairy cows	75 mg of FBs/kg and 3 mg FB_1_/kg BW	none	[185]	
FB_1_	ET	Steers	94 mg FB_1_/kg diet	↑ AST, GGT, hepatocellular injury and biliary epithelial hyperplasia	[23]	Exposition period of 253 days
FBs	ET	Claves	15, 31 or 148 mg FBs/kg diet	= Feed ingestion, BW ↑AST, GGT, LDH, bilirubin and cholesterol	[186]	
FB_1_	ET	Milk-fed calves	1 mg of FB_1_/kg BW intravenous administered	Liver and kidney lesions ↑ serum AST, ALP, GGT and sorbitol dehydrogenase	[187]	
FBs	ET	Lambs	0, 11.1, 22.2 or 45.5 mg of FBs/kg BW	Death, ↑ alkaline phosphatase, GGT, AST, cholesterol, triglyceride, urea nitrogen and creatinine	[188]	
ZEA	ET	Heifers	250 mg ZEA/heifer	↓ Conception rate, no other effects	[189]	
ZEA	ET	Dairy cow	from 0 to 500 mg ZEA/cow	None	[190]	
DON & ZEA	FT	Heifers	About 500 μg of DON/kg diet and 750 μg of ZEA/kg diet	unsynchronized ovarian cycles, vaginitis and early development of mammary gland in the prepubertal heifers	[191]	
ZEA	ET	Ewes	1.5, 3, 6, 12, or 24 mg ZEA/ewe	reproductive disorders, lower lambing percentages and infertility.	[192]	
OTA	ET	Sheep	0, 1.4, or 3.5 mg of OTA/kg diet	=feed intake and nutrient utilization	[193]	
OTA	ET	Sheep	14 mg of OTA/kg diet	↓ feed intake	[193]	Preliminary ET
Mychopenolic acid	ET	Sheep (male)	0, 10, 70, 300 mg of MPA/sheep/day	none	[194]	
Mychopenolic acid	ET	Sheep	300 mg of MPA/sheep/day	Slightly signs of immunosuppression in jejunum, white blood cells, ileum	[195]	
Mychopenolic acid	ET	Sheep	300 mg of MPA/sheep/day	none	[196]	
Roquefortine C	FT	Cow	about 4–8 mg of RC/kg diet	Reversible paralytic effects	[197]	
Roquefortine C	ET	Sheep	0, 10 and 50 mg of RC/sheep/day	None ↓ rumen pH	[95]	
Citrinin	FT	Sheep	Presence of visible moldy feeds in diets contaminated by both citrinin (2–10 mg/kg) and OTA (0–20 mg/kg)	fever, diarrhea and uraemia	[198]	
Citrinin, monacolin K, pravastatin and mevastatin	ET	Sheep	*Monascus* fermented rice	None ↓ rumen methane production	[154]	
Patulin	FT	Beef	Suspected Patulin	neurotoxicosis, comprising tremors, ataxia, paresis, recumbency and death	[199]	

^a^: aflatoxin B_1_, AFB_1_; ammonia nitrogen, NH_3_-N; average daily gain, ADG; average daily intake, ADI; average daily weight, ADW; aspartate aminotransferase, AST; body weight, BW; deoxynivalenol, DON; diacetoxyscirpenol, DAS; dry matter intake, DMI; dry matter, DM; forage to concentrate ratio, F:C; fumonisin B_1_, FB_1_; fumonisin B2, FB2; γ-glutamyltransferase, GGT; lactate dehydrogenase, LDH; Mychopenolic acid, MPA; ochratoxin A, OTA; Roquefortine C, RC; volatile fatty acids, VFA; zearalenone, ZEA.

In our opinion, the lack of unequivocal information regarding mycotoxin effects on ruminants should be related to the complexity to plan specific animal trials since a multitude of confounding effects exist. Among these, there are: (1) effect of mycotoxin on cattle and other ruminants depends by several factors, such as toxin-related (type and level of mycotoxin ingested as well as duration of intoxication period), diet-related (inclusion level of mycotoxin contaminated feeds, diet composition, forage to concentrate ratio, diet physical form, digestibility of dry matter or other nutrients, rate of passage, *etc.*), animal-related (species, sex, age, breed, dry matter intake level, general health, immune status, nutritional strategies) and environmental-related (farm management, hygiene, temperature, *etc.*) factors [134]; (2) for feeding experiments, it is strongly recommended to feed animals a known quantity of mycotoxins and to monitor individual daily mycotoxin intake because the main objective of these types of trials is to clarify the effect of one or at least few mycotoxins. However, feeds may be contaminated by more than one known and several unknown or unchecked mycotoxins. The toxic responses and clinical signs observed in animals ingesting multiple-contaminated feeds are more complex and diversified with respect to animals assuming feeds contaminated by one/two mycotoxins (rare) or their chemical standards (unrealistic). In particular, when mycotoxins are present simultaneously, some interactive effects, classified as additive, antagonistic or synergistic, could occur [18,200,201]. For instance, in the CAST report [4], authors reviewed 33 studies on mycotoxin interaction effects in farm animals, indicating that additive or antagonist effects were the predominant effects (78%). However, only two studies were carried out on ruminants, lambs in particular [164,184]; (3) mycotoxins can be modified mainly by plant and conjugated with polar compounds such as glucose, malonic acid and glutathione [124,126]. Modified mycotoxins are produced via enzymatic transformations related to plant detoxification processes and have been related to a resistance mechanism to counteract pathogen invasion [118,123,124,125]. Up to now, little is known about bioavailability of modified forms of mycotoxins, beyond DON and to some extent ZEA [127]. Evidences suggest they can be hydrolyzed and absorbed in the gastrointestinal tract of animals thus contributing to the overall exposure. Based on the few data currently available, the modified forms of a mycotoxin probably exert the same toxicity as the parent compound and when assessing the toxicity of modified mycotoxins it is important to determine the percentage of modified mycotoxin hydrolyzed in the intestinal tract [127].

### 3.1. Alternaria Derived Toxins

On 2011, the European Food Safety Authority (EFSA) reviewed information regarding safety of *Alternaria* derived toxins in food and feed, such as alternariol, alternariol monomethyl ether, tenuazonic acid, iso-tenuazonic acid, altertoxins, tentoxin, altenuene and AAL-toxins [74]. Generally, alternariol and alternariol monomethyl ether are genotoxic for bacteria and mammalian cells *in vitro*, whereas altertoxins are mutagenic for bacteria and induce cell transformation. Tenuazonic acid and tentoxin are not mutagenic for bacteria [202,203]. As clearly stated in the EFSA scientific opinion [74], the estimation of intake levels was limited to chicken, the only species for which some toxigenic data suitable for risk assessment exist. No information about exposure and toxicity due to *Alternaria* derived toxins were currently available for livestock, ruminants in particular. Consequently, information on susceptibility of farm animals to *Alternaria* derived compounds is needed, as these are largely detected in food and feeds [48,204,205,206,207]. The EFSA report [74] has been seriously questioned especially with respect to AAL toxins, numerous undocumented claims being found [72].

### 3.2. Aspergillus Derived Toxins

AFs as group are considered the most potent carcinogenic natural substances and have been classified as group 1 carcinogens by International Agency for Research on Cancer [208]. When ingested, AFs are rapidly adsorbed in the gastro-intestinal tract and quickly appear as metabolites in blood just after 5 min [209] and in milk at first milking [139,169,210,211]. The principal oxidized metabolite of AFB_1_ (*i.e.*, AFM_1_) can be found in milk of lactating animals, thus representing a risk for human health. Consequently, ingestion safety levels for AFB_1_ in lactating dairy cows should be assessed on carry over rate of parent molecules into milk as a function of specific legislation [169,210,212]. Mechanism of action, toxic properties, human and animal exposures to AFs ingestion and risk due to milk contamination were extensively reviewed [4,139,213,214,215,216,217]. In the rumen, AFB_1_ is converted to aflatoxicol, AFM_1_ and many other hydroxylated metabolites [3,31,218,219] or sequestered by different rumen fluid components such as chlorophyllin structures, bacteria and yeast cell walls [29,30,219,220]. Despite the protection activity of rumen fluid, *in vitro* studies [141,144] suggested presence of increasing AFB_1_ levels in rumen fluid reduced gas production, ammonia N and VFA concentrations, showing therefore an antibacterial activity. Anyway, a hypothetical AFB_1_ diet concentration ranging from about 650 to 2000 μg/kg DM, estimated by considering a fixed rumen fluid volume of 50 L and a DMI of 23.5 kg/cow/day, could be calculated from AFB_1_ doses tested in these works. Similarly, Westlake *et al.* [142] showed presence of AFB_1_ in rumen fluid drastically reduced rumen digestibility of alfalfa by about 50% and 67% at 1 and 10 μg AFB_1_/mL buffered rumen fluid, respectively. Conversely, Auerbach *et al.* [143] reported a rumen AFB_1_ content of 9.5 ng/mL did not modify *in vitro* digestion of alfalfa and VFA productions. Consequently, the amount of AFs affecting animal performance and impairing their health is much greater than the dietary amounts associated with milk residues [134,169]. However, sheep [164,165,166,167] and dairy cows [169] exposed to AFs-contaminated diets reduced ingestion and presented alteration of hepatic activity and immune-suppression also at relatively low levels of mycotoxin ingestion. Furthermore, replacement heifers exposed to diets co-contaminated by AFs and FBs at increasing levels showed an important delay in reproductive career along with a slow growth [170].

Among about 500 cases submitted for necropsy at the Department of Pharmacology and Pathobiology of the Royal Veterinary and Agricultural University of Denmark from 1987 to 1992, 30 were diagnosed as Aspergillosis caused by *A. fumigatus* and zygomycosis by fungi of the class Zygomycetes [129]. The main target organs for invasive fungal infection were omasum followed by the rumen-reticulum and abomasum. Furthermore, Frisvad *et al.* [92] reported *A. fumigatus* produces several metabolites with antimicrobial, antifungal or antiprotozoal effects. Among these, gliotoxin is immunosuppressive, causing apoptosis of lymphocytes and macrophages or ROS could be generated and could cause damage to healthy cells in the organs of the host [8,221,222]. In CAST report [4] were summarized several observations relating presence of this mycotoxin to pathogenesis of Aspergillosis. Anyway, evidences supporting gliotoxin is the cause of mycotoxicosis in livestock are unverified and, as discussed previously, it is not likely to be formed in cereals. Concerning the antimicrobial effects of gliotoxin, Morgavi *et al.* [149] stated that only very high levels of gliotoxin, up to 80 μg/mL of rumen inoculum, influenced DM degradation, gas and VFA productions. Following same static assumptions reported above (50 L rumen fluid volume and 23.5 DMI), the level of 80 μg/mL of rumen inoculum results in a diet contaminated by a gliotoxin level of 140 mg/kg DM, three times higher than what expected by using in dairy cow diet the highest gliotoxin contaminated MS found by Pereyra *et al.* [81]. Anyway, an extract containing 8.8 μg of gliotoxin/mL decreased DM degradation, gas and VFA productions by 28%, 46% and 35% [149]. On farm conditions, *A. fumigatus* has been proposed as the pathogenic agent associated with mycotic haemorrhagic bowel syndrome in dairy cattle cases occurring in US and often attributed to *Clostridium* infections [137,223]. However, this piece of information was not supported by scientific evidences.

Other toxic compounds produced by *Aspergillus* strains are kojic acid, β-nitropropionic acid and cyclopiazonic acid [84]. Report of US Environment Protection Agency [172] stated these toxins possess antibacterial and antifungal activities. In particular, maltoryzine was associated with poisoning in dairy cows, but this information was not supported by references. Cyclopiazonic acid is toxic for several animal species and causes disruption of calcium homeostasis, degeneration and necrosis of the liver, lesions of myocardium, degeneration or death of cells and neurotoxins effects [224]. Anyway, all experiments reporting these effects were carried out on monogastric species [4]. Last, β-nitropropionic acid is a neurotoxin and its mode of action is an apparently irreversible succinate dehydrogenase inhibition [172]. Chronic or acute intoxications by β-nitropropionic acid on sheep and cattle [173] caused emphysema and difficulty in locomotion. Furthermore, microscopic lesions in the lungs, cells of central nervous system and Wallerian degeneration of the spinal cord were reported. No other information on livestock are currently available for this compound.

There is an extensive literature on the toxicokinetics, metabolism and tissue distribution of OTA [225]. In ruminants, OTA is largely degraded by ruminal microflora into the less toxic ochratoxin α [3,8,31,131,226,227] due mainly to the activity of protozoa [33,151,228,229]. In young calves, more than 90% of orally administered OTA is excreted in urine as metabolite ochratoxin α [230]. Blank *et al.* [231] investigated the metabolism of OTA feeding sheep 0, 9.5, 19.0 and 28.5 μg OTA/kg BW. Serum concentrations of OTA increased with exposition levels of animals and small amounts of ochratoxin α were detected in plasma, suggesting OTA could bypass rumen undegraded [27,232]. Similar results were reported by Höhler *et al.* [193] fed sheep 0, 1.4, or 3.5 mg of OTA/kg diet, even if no effects on feed intake and nutrient digestibility were reported. However, in a preliminary trial, the same authors reported sheep fed 14 mg of OTA/kg diet reduced feed ingestion. Even though OTA can escape ruminal degradation and traces were found in milk of experimentally exposed ewes, Boudra *et al.* [233] concluded the low carryover of OTA in milk minimizes the risk for consumers. Ribelin *et al.* [234] indicated that the lethal single oral dose of OTA in cattle is probably higher than 13 mg/kg of BW, but not recent upgrades have been reported.

Niederberger *et al.* [235] reported 5 heifers from one farm in Germany were affected by muscular tremor, hyperexcitability and hypersensitivity. Histological examination of animals revealed degeneration of neurons in the brainstem. Analyzing silage, presence of *Aspergillus clavatus*, a mold capable of producing neurotoxic tremorgenic mycotoxins, patulin and maltoryzin [236], was detected.

### 3.3. Fusarium Derived Toxins

The toxicological effects of *Fusarium* derived toxins in farm animals are deeply described [2,25,26,216,237,238]. DON and other trichothecenes, such as T-2 and HT-2 toxins, DAS and nivalenol, have been suspected to be implicated in farm animal disease outbreaks in many areas of the World [239]. Generally, trichothecenes type B are considered to be more toxic than type A for ruminants [83]. The number of ascertained cases of intoxication by *Fusarium* derived toxins remains low on field conditions, being this toxicosis often characterized by non-specific clinical symptoms [3,25,240].

Although DON is not suspected to cause acute toxicity in ruminants, it is considered to be the major cause of economic losses due to reduction of animal performance [241]. Clinical signs due to contaminated DON feed ingestion include gastrointestinal problems, soft stools, diarrhea, immunosuppression and a general decrease of performances probably due to feed refusal [3,242]. Generally, dairy cattle are retained more sensitive to the effects of DON compared to beef cattle and sheep [134]. Charmley *et al.* [176] carried out an experiment to determine the effect of DON on cow performance. The increasing daily intakes of DON were 0.59, 42, and 104 mg/cow/day. However, no effects were measured on intake and milk production of lactating animals. Only milk fat was drastically reduced (lowest value for intermediate treatment). Trenholm *et al.* [178] reported no lactating dairy cows consuming a wheat-oat DON-contaminated concentrate (1 kg/100 kg BW with a DON contamination of 6.4 mg/kg) slightly reduced ingestion of feed, even if no signs of illness as well as BW gain decrease were recorded. Similar absence of signs was reported by other authors [177,182,243]. Dänicke *et al.* [179] reported an increase in rumen ammonia concentration and a reduction in duodenal flow of microbial protein feeding rumen-duodenal fistulated no lactating dairy cows with a *Fusarium* toxin contaminated (DON and ZEA) wheat. The influence of DON on fermentation parameters, in particular on interruption of microbial protein synthesis or alteration of pH, was successively confirmed by Jeong *et al.* [147] carrying out an *in vitro* trial and by Keese *et al.* [174,175] directly on lactating dairy cows.

Other *in vitro* data [145,146] indicated the incubation of DON (5 mg of DON/kg diet) and other *Fusarium* toxins (ZEA, nivalenol, scirpentriol, 15-acetyldeoxynivalenol, and 3-acetyldeoxynivalenol) in diluted rumen fluid did not alter normal fermentation activity of rumen inocula, except for the activity of cellulosolitic bacteria. Korosteleva *et al.* [180,181] reported *Fusarium* contaminated diets (3.5 mg of DON/kg diet and 0.24 mg of ZEA/kg diet) affected metabolic parameters and immunity of lactating dairy cows, even if no effect on DM intake or milk performance was reported. Kiyothong *et al.* [171] reported lactating dairy cows fed a diet naturally contaminated with AFB_1_ and several *Fusarium* derived toxins showed lower DMI and nutrient digestibility than cows fed the same diet supplemented with a mycotoxin deactivating product. Furthermore, both hematological and immune parameters were adversely affected in cows receiving contaminated diet without product supplementation. Consequently, the impact of DON ingestion in lactating dairy cows is still controversial and needs future clarifications. These controversial results could be attributed to a different rumen activity in converting DON parent molecula into less toxic de-epoxidized metabolites [31,135,244]. Last, fusaric acid and DON were tested for antimicrobial activity against *Ruminococcus albus* and *Methanobrevibacter ruminantium*. The growth of both organisms was inhibited by fusaric acid but not by DON and consequently no synergistic inhibitory effect was observed [148].

Concerning health hazard due to ingestion of nivalenol, Hedman and Pettersson [245] reported ruminal microbiota was able to produce a de-epoxidised metabolite of nivalenol, thus suggesting a possible detoxification mechanism. Despite in EFSA scientific report [246] nivalenol exposure levels for lactating dairy cows and beefs are described, no information about the effects of its ingestion in livestock are currently available. A similar lack of information is present for fusarenon X.

Concerning trichothecenes type A, the adverse effects due to ingestion of diet contaminated by T-2 and HT-2 toxins have been extensively reviewed [247], but the majority of researchers carried out studies between 70s to 80s and they tested effects of trichothecene mycotoxins on young ruminants (in particular calves and lambs) [248,249,250,251,252,253]. The main effects referred to hemorrhages and lesions in the gastrointestinal tract, enteritis or bloody feces as well as changed in metabolic and immune status of animals. Effects of T-2 and HT-2 on semen quality have been suspected in bulls [254]. To the best of our knowledge, no information are currently available for lactating dairy cows or beef. Accordingly, the ESFA scientific report [247] concluded saying exposure level equal or higher than 0.3 mg T-2 toxin/kg BW per day may result in gastrointestinal lesions, altered serum proteins and hematological alterations in calves or lambs, whereas the limited data on lactating dairy cows do not allow to set a safety level of ingestion.

Concerning DAS, an experience was reported by Harvey *et al.* [184]. In this trail, lambs were fed for 14 days with control (uncontaminated), AFB_1_-contaminated (2.5 mg/kg), DAS-contaminated (5 mg/kg from chemical standard) and AFB_1_/DAS co-contaminated (2.5 mg of AFB_1_ and 5 mg of DAS/kg) diets. Animals receiving contaminated diets reduced feed ingestion by 7% to 12% thus probably causing a decrease in BW during intoxication period (difference between initial and final BW of 0.1, −0.6 and −2.7 kg for AFB_1_-, DAS- and AFB_1_/DAS-contaminated diets, respectively).

Among *Fusarium* derived toxins, FBs are cytotoxic, hepatotoxic and nephrotoxic to animals, even if mechanism of action is not completely elucidated [4,135,216,255]. Furthermore, they are inhibitors of cellular sphingosine (sphinganine) *N*-acetyltransferase that resulted in accumulation of sphinganine and sphingosine and a depletion of complex sphingolipids in eukaryotic cells, which in turn results in impairment of cell cycle regulation, cellular differentiation and in oxidative stress as well as apoptosis and necrosis [256]. In contrast to many other mycotoxins, FBs are poorly degraded in rumen compartment [31,150,257]. Major clinical signs of FBs poisoning in livestock are decreased appetite accompanied by serum biochemical and histologic evidences of hepatic damage. However, lactating dairy Jersey cows fed a diet contaminated at a level of 75 mg/kg as well as two cows consuming 3 mg FB_1_/kg BW did not show any clinical or hematological changes. Only transient diarrhea at the beginning of intoxication period and an increase in serum cholesterol were reported [185]. In a successive experiment, Holstein steers were fed a diet with a contamination level of 94 mg FB_1_/kg for 253 days and increases in serum aspartate aminotransferase (AST) and γ-glutamyltransferase (GGT) as well as hepatocellular injury and hyperplastic biliary epithelial cells were reported [23]. Likewise, peripubertal heifers fed diets contaminated by high levels of both AFs and FBs changed some parameters of plasma metabolic profile [170]. Similar metabolic changes, such as serum increase of AST, GGT, lactate dehydrogenase (LDH), bilirubin or cholesterol and histological changes were reported when calves were fed diets containing 15, 31 or 148 mg FBs/kg diet for 31 days [186]. However, no effects were measured on feed intake or weight gain, even if feed containing the highest FBs level seemed to be less palatable. At the highest dose, lymphocyte blastogenesis was significantly impaired at the end of intoxication period. To examine the effects of acute exposure to FBs, lambs were intraruminally dosed with increasing levels of FBs from *Fusarium verticillioides* culture material. The treatments were 0, 11.1, 22.2 or 45.5 mg of FBs/kg BW for 4 days and death occurred in the two highest dose groups [188]. For survival animals, increases in alkaline phosphatase, GGT, AST and LDH activities as well as in cholesterol, triglyceride, urea nitrogen and creatinine levels were observed. Furthermore, histological examination at the end of the trial revealed renal tubular necrosis and a mild hepatopathy.

The ZEA is converted in rumen compartment into two hydroxyl-metabolites, being α-zearalenol (α-ZOL) and β-zearalenol (β-ZOL) [131,258], with about 90% of parent molecules converted into α-ZOL [31]. The α-ZOL is more oestrogenic than parent molecula but it is slowly absorbed in the liver and could be converted by this organ to the less potent β-ZOL [138]. About effects of ingestion of ZEA-contaminated diets in livestock, experimental studies are lacking, but some case reports indicated that after exposure to high doses of ZEA, animal could present reproductive problems, such as decrease in embryo survival, edema and hypertrophy of the genitalia in pre-pubertal females, decrease in production of luteinizing hormone and progesterone, changes in morphology of uterine tissues, feminization of young males due to decrease of testosterone production, and more generally infertility [2,134,138,259]. Either mode of action and toxicological studies of ZEA were reviewed [260]. The effects of ZEA were studied on heifers [189] and dairy cows [190]. In both studies, pure ZEA (250 mg/heifers and from 0 to 500.0 mg/no pregnant dry cows) was orally administered to animals. The only effect measured was a lower conception rate in treated heifers with respect to control. No effects on the reproductive organs and no changes in the progesterone blood concentrations were detected. In a dairy herd, animals receiving a diet contaminated with both DON and ZEA at levels of about 500 and 750 μg/kg, respectively, showed unsynchronized ovarian cycles, vaginitis and early development of mammary gland in heifers [191]. However, heifers fed a diet with a ZEA concentration of 1.25 mg/kg diet did not show reproductive problems. In addition, several field or case reports in which a direct relationship between ZEA exposition levels and symptoms of estrogenic effects was not found were reported [260], suggesting this might reflect the variability in rumen degradation of ZEA. Recent experiences tried to relate exposure of dairy cows to ZEA contaminated diet on herd level by measuring urinary metabolites [261,262,263]. Authors suggested that monitoring urinary ZEA concentrations could represent an useful tool to predict animal exposure to ZEA and other *Fusarium* toxins. Smith *et al.* [192], feeding ewes with increasing ZEA level (1.5, 3, 6, 12, or 24 mg ZEA/ewe), measured reproductive disorders, lower lambing percentages and infertility. At the highest doses, increases in oestrus duration or uterus and ovarian weights were observed too. Fink-Gremmels and Malekinejad [25] reported α-ZOL is used in many Countries as growth promoting agent in fattening cattle and lambs.

No information are currently available concerning effects of other *Fusarium* derived toxins, such as beauvericins, enniatins and moniliformin [8,206].

### 3.4. Penicillium Derived Toxins

*P. roqueforti* and *P. paneum* produce several secondary metabolites with immunosuppressive, antibacterial and other not well-defined toxicological effects for animals [3,8,11,24,130]. Insufficient and controversial information have been reported concerning effects of these mycotoxins on animals. Furthermore, different authors referred feeding forages contaminated by *Penicillium* strains can cause loss of appetite and impact nutrient efficiency, increase in somatic cell counts, ketosis, abomasal ulcer, laminitis, gastroenteritis, paralysis and abortion, probably due to the production of their toxic metabolites [3,11,128,195,264,265]. However, no adverse effects on animal health and blood parameters were detected in sheep fed 300 mg/day of mycophenolic acid [194]. Recent experiences carried out by Dzidic *et al.* [195,196] indicated that sheep fed 300 mg of mycophenolic acid/sheep/day from contaminated silage did not show any immunodepression effects. Furthermore, *Penicillium* derived toxins such as citrinin, OTA, patulin, mycophenolic acid, penicillic acid or a combination of one of these mycotoxins with OTA could inhibit activity of macrophage up to 25%, thus confirming immunomodulatory properties of these toxins and possible increase of the risk of disease susceptibility in cattle consuming contaminated diets [266].

Santos and Fink-Gremmels [16] verified the effect of ingestion of moldy silages on cows by the individuation of several biomarkers helpful for characterizing mycotoxin syndrome in cattle. In particular, three farms were selected on the basis of the clinical diagnosis of the local veterinarians who observed that animals showed loss in body condition score, poor feces consistence, signs of lameness without a clear disease condition and an irregular increase in somatic cell counts along with an unexpected low milk yield. The GS fed to animals in these farms were sampled and analyzed for mycotoxin contaminations. All these silages resulted highly contaminated by mycophenolic acid and by others *Aspergillus* (*i.e.*, cyclopiazonic acid, gliotoxin and β-nitropropionic acid) or *Fusarium* (*i.e.*, DON, ZEA, FB_1_) derived mycotoxins. The use of biomarkers to verify mycotoxicosis exposure for humans has been proposed mainly for regulated mycotoxins [267,268,269,270,271]. On the other hand, Santos and Fink-Gremmels [16] selected specific markers of oxidative stress, lipid metabolism and liver function for monitoring mycotoxin effects in lactating dairy cows. In these specific conditions, oxidative stress as well as a dysfunction of lipid metabolism were observed in animals ingesting these moldy silages. Effects measured into blood of animals were decreases of glutathione peroxidase activity level, glucose-6-phosphate-dehydrogenase concentrations, trolox equivalent antioxidant capacity, activity of phospholipid transfer protein and lecithin-cholesterol acyltransferase along with an increase in free cholesterol concentration. We retained this approach could be useful to clarify effects of mycotoxins on livestock. Consequently, these aspects should be further investigated to improve understanding of the pathophysiological changes associated with the multiple mycotoxin exposure in dairy cows, thus allowing for refined assessment of intervention strategies.

An *in vitro* trial was carried out by Gallo *et al.* [130] to verify effects of some *Penicillum* derived mycotoxins (*i.e.*, mychopenolic acid, roquefortine C and PR toxin) on rumen fermentation parameters as well as to assess their stability in the rumen environment. Mycotoxin doses ranging from 0.1 to 2 μg/mL rumen fluid or from 0 to 2 μg/mL rumen fluid were tested in two successive trials. The concomitant presence of mycophenolic acid, roquefortine C and PR toxin in trial 1 or only mycophenolic acid and roquefortine C in trial 2 was tested to verify combined/synergic effects of these mycotoxins. Both mycophenolic acid and roquefortine C influenced curve parameters and decreases final gas production of about 13%–15% at the highest concentrations. These mycotoxins, extracted from highly-contaminated MS (contaminations higher than 10 mg/kg for at least one of two mycotoxins), had 30%–40% higher depressing effects on gas and VFA production than those predicted by the model developed by using pure mycotoxins (Gallo, data not reported). These findings suggested other secondary metabolites or the release of bound (modified) mycotoxins during incubation could worsen the effect of these toxins on rumen microorganisms. Furthermore, the stabilities of these two toxins after 48 h of rumen fluid incubation were similar and on average equal to about 50%. PR toxin did not interfere with rumen fermentation pattern and it was not detected after 48 h of incubation. Consequently, it was verified that mycophenolic acid and roquefortine C from standards additively interfered with rumen microorganisms at relatively low levels and were stable in rumen environment after 48 h of incubation, suggesting these mycotoxins could interfere with digestive processes and might represent a potential risk for ruminants. *In vivo*, reversible paralytic effects were reported in cows that ingested *P. roqueforti*-contaminated feed grains containing an average roquefortine C concentration of 25.3 mg/kg [197]. Tiwary *et al.* [272] reported roquefortine C did not appear to be responsible for tremorgenic effects and could be quantified as biomarker for penitrem A exposure. Tüller *et al.* [95] feeding sheep 0, 10 and 50 mg/sheep/day of roquefortine C did not report any effects on chemical and hematological parameters. However, the rumen pH decreased of 0.5 after intoxication. Lastly, even if PR has been suspected to be associated with cattle disorders [137,273], effects due its ingestion by farm animals have not yet been thoroughly investigated.

Patulin is produced by *P. paneum* as well as *B. nivea*. Patulin drastically interfered with rumen activity [152,153], even if effects of its ingestion on ruminants are actually unknown. Only Sabater-Vilar *et al.* [199] reported severe cases of neurotoxicosis, comprising tremors, ataxia, paresis, recumbence and death concomitantly occurred in several herds of beef cattle in Belgium. As described by these authors, *Aspergillus clavatus* was found to be the dominant fungal species in a feed containing malting residues and consumed by all these herds. The isolated fungus produced patulin in culture medium and mycotoxixosis caused by this toxin was suspected. For either andrastins nor marcfortines, no information have been reported regarding their effects on livestock and their fate in rumen compartment [8].

### 3.5. Monascus Ruber Derived Toxins

Citrinin is a nephrotoxic mycotoxin produced by several species of the genera *Aspergillus* and *Penicillium* [274]. Citrinin can occur also as an undesirable contaminant in *Monascus ruber* fermentation products and concomitant occurrence of OTA in food or feed materials has been often reported [274,275]. Field experiments [198,276] suggested cows fed citrinin-contaminated diets showed signs of pruritus, pyrexia and hemorrhagic syndrome as well as fever, diarrhea and uremia. In these experiences, animals ingested visible moldy feeds contaminated by both citrinin (30–40 μg/kg or 2–10 mg/kg) and OTA (0–20 mg/kg). Based on *in vitro* results, Stec *et al.* [277] reported immunotoxic effects of citrinin only at very high doses. Experimental data regarding systemic toxic effects in ruminants were not available and it is assumed that citrinin is highly degraded and metabolized through the microbial activity in the forestomachs of ruminants [274]. However, an impairment of the rumen microflora due to the antibacterial effect of citrinin cannot be excluded. Recently, Morgavi *et al.* [154] verified that the antimethanogenic activity of metabolites produced from different *Monascus* spp., such as monacolin K, pravastatin, mevastatin and citrinin. These substances showed an inhibitory effect on methanogens thus decreasing methanogenesis *in vitro* and in short term *in vivo* studies, without affecting rumen fermentation pattern.

No toxin effect has been associated with the consumption of monacolins contaminated diets in farm animals [8].

### 3.6. Endophytic Fungal Toxins

Grasses have relatively few intrinsic toxins, relying more on growth habit to survive defoliation and endophytic fungal toxins as chemical defenses [5,57]. Endophytic toxins in grasses include ergot alkaloids in tall fescue and tremorgens in perennial ryegrass [137,223]. Although a number of tremorgens have been identified, the most important is lolitrem B, produced by endophytic fungus. Lolitrems cause neurological effects, producing the ryegrass staggers syndrome [3,4]. Cattle consuming tall fescue contaminated with endophytic fungi have also shown symptoms of stumbles, excitability, increased of rectal temperature and respiration rate as well as decrease in BW [1].

### 3.7. Intestinal Modulation of Mycotoxins

Fink-Gremmels [31] reported some mycotoxins could pass rumen unchanged (cyclopiazonic acid, FBs, patulin), almost completely metabolized in the rumen in less toxic compounds (OTA in ochratoxin-α, DON in de-epoxy-DON, ZEA in β-ZOL, AFB_1_ in AFM_1_) or in rumen compartment in converted in metabolites with similar or higher toxic activity than parent molecules (ZEA in α-ZOL, AFB_1_ in aflatoxicol). Consequently, rumen could have a great capability to inactivate mycotoxins and reduce health risk in cattle for some mycotoxins, whereas for others it results completely inefficient in protecting animals by negative effects due to mycotoxin ingestion. The protective effect of rumen could be compromised when health status of animals is altered, for any changes in diet composition or as function of mycotoxin exposition levels [31,217]. At the same time, different *in vitro* trials reported mycotoxins such as AFB_1_ [29,218], DON [147], gliotoxin [149], FBs [31], OTA [151] or mycophenolic acid and roquefortine C [130] resulted partially stable in rumen environment and thus could reach intestine unchanged with possible antimicrobial activity on intestinal microflora or toxic effects on host animals. Furthermore, Fink-Gremmels [31] and Flores-Flores *et al.* [217] recently summarized information concerning mycotoxin milk contamination with the aim to evaluate possible risk for humans. Other than AFM_1_, several mycotoxins such as other AFs (AFG_1_, AFG_2_, AFB_1_, AFB_2_, AFM_2_), cyclopiazonic acid, FB_1_, nivalenol, OTA and ochratoxin α, T-2 toxin, ZEA and its metabolites or DON and its de-epoxy metabolite have been found in milk and we refer to these reviews for extensively discussions of these aspects.

Generally, maintenance of a healthy intestine is crucial to assure adequate nutrient absorption, maintenance of the indigenous microflora and protection of host animals against pathogens thus guaranteeing a correct function of immune system. However, studies on the effect of these mycotoxins on the gastrointestinal tract are limited, in particular for ruminants. Several authors [18,239,278,279] reviewed how different mycotoxins such as AFs, OTA, DON, T-2 toxin, ZEA and FBs, impact digestive and absorptive functions, intestinal defences and intestinal microbioma composition. In particular, *Fusarium* derived toxins, mainly DON and FB_1_, could drastically alter the defences mechanisms of intestine, reducing epithelial integrity, cell proliferation and mucus production or increasing intestinal permeability, immunoglobulins and cytokine productions [239]. Data from many research studies carried out on monogastric animals showed that mycotoxins can compromise several intestinal functions, such as digestion, absorption, permeability, defences and can result in lower productivity and poor health of animals [239,278,280]. However, experiments elucidating the effects of mycotoxins on intestinal functionality as well as interference with intestinal microbiota are absent for ruminants and need to be verified in the future, even because the rumen could produce known and unknown mycotoxin metabolites absent in monogastric diets.

## 4. On Farm Strategies to Minimize Risk of Mycotoxin Contaminations in Forages

### 4.1. Prevention of Mycotoxin Contaminations of Crops in Field and during Storage

Generally, mycotoxin contamination of agricultural products should be prevented or counteracted by using pre-harvest or post-harvest strategies. Several strategies have been investigated to avoid mycotoxin occurrence in each ring of the food chain. The simplest strategy is based on the prevention of mycotoxin formation in feeds. At field level, different steps could be effective to prevent fungal infestation and consequently mycotoxin production. Among field actions, the most important to counteract fungal infestations are: opportune crop rotation, tillage, soil fertilizers, planting date, crop hybrid/variety selection, chemical/biological control of infestation, crop removal, insect and weed controls. These aspects were widely discussed [22,64,281,282,283,284,285,286].

Under farm conditions, the storage of crops represents another critical step [285]. In particular, grains should be preserved for physical integrity and properly stored, with a moisture content lower than 13% and at low temperature [6,287,288,289]. Despite all precautions, it may happen that stored grains could be damaged and infected by molds and probably by mycotoxins. As recommended by Jard *et al.* [285], the farmers must discard moldy grains and any material that is suspected of being contaminated with mycotoxins, including apparently clean grains in the vicinity of moldy parts.

Concerning silages, a low oxygen concentration and augmentation of carbon dioxide are efficient in preventing mold development [290]. Consequently, all ensiling stages, such as aerobic, fermentation, stable, feed-out or aerobic spoilage phases should be controlled and optimized as much as possible to assure adequate conservation of ensiled crops [40,290]. However, ensiling procedures are not standardized and farmers use different procedures to ensile and store silages [69,120]. As extensively reviewed by Muck [291] and Dunière *et al.* [140], uncorrected silo management conditions, such as inappropriate DM content of crop at harvest for its effect in influencing final silage packing density, inadequate particle length, slow silo filling, imperfect mass sealing, poor mass compression, delay in mass pH drop, air penetration in ensiled mass or inappropriate unloading equipment and techniques, could compromise any of aforementioned ensiling phases, thus exposing silages to risk of air penetration and consequent activity of aerobic spoilage microorganisms [68,292,293]. In particular, aerobic deterioration could cause nutrient and DM losses, heat damage of nutrients, excessive proteolysis, proliferation of undesirable microorganisms, such as mycotoxigenic fungi, and production of their toxins [82]. The negative effects due to aerobic activity could be more serious in specific areas of silage, especially in the peripheral (both lateral and apical) parts of ensiled crop, which are generally packed and sealed with difficulty [67,68,69]. Furthermore, when silo is opened for feeding, oxygen becomes available to the front of the mass and the activity of the yeasts and molds, as a result of survival of fungal spores or a re-colonization of these microorganisms, could reduce aerobic stability of ensiled mass, thus favoring potentially toxigenic fungi development [28,36,68,264]. On farms, the adoption of correct ensiling procedures enables to reduce the area exposed to risk of air penetration, such as proper humidity of crops at harvest, use of additives, proper particle sizes, adequate silo size, optimal mass compression, use of polythene wall sheet or different polythene sheets covering ensiled mass, uniform and adequate distribution of weight on the top of ensiled mass limiting oxygen contact in peripheral zones of silo, rapid progress through the silage face, represent the best strategies to guarantee the safety and fermentative quality of ensiled crops [69,140]. Alternative ways for improving or guaranteeing the aerobic stability of silages consist in applying acid-based additives [294]. However, the use of such type of additives may result expensive [295] and the efficiency in the improvement of aerobic stability has not been sufficiently demonstrated [296]. Microbial inoculants such as lactic acid bacteria (LAB) are currently used as economical and practical alternatives to acid-based additives [295,297]. The use of beneficial microbial inoculants to silages before ensiling could improve fermentation occurring during all ensiling phases. However, homofermentative LAB, such as *Lactobacillus plantarum*, can produce a silage that is poorly stable when exposed to air, because the low production of antifungal compounds such as acetic acid [295,298,299]. Even if heterolactic fermentation is less efficient in the conservation of nutrients than homolactic fermentation [295], the use of heterolactic LAB inoculants, such as *L. buchneri*, has showed the potential to improve the production of silage from easy, moderately difficult and difficult to ensile materials by reducing the pH, ammonia nitrogen and DM losses [295,297,300]. This because a high production of acetic acid, with a higher antimycotic activity than lactic acid, occurs [69,295].

To obtain information about the rate and extent of either favorable or adverse fermentations that occur in silages, fermentation end-products are commonly used. To this end, different fermentative quality indexes, such as Flieg-Zimmer’s or Vanbelle-Bertin’s scores [301], have been proposed to rank between well- and poorly preserved forages according to the relative amounts of lactic acid, acetic acid, butyric acid or ammonia nitrogen. Recently, Gallo *et al.* [69,120] developed an index by using a multivariate approach (factorial analysis) to evaluate fermentative quality of MS. This fermentative quality index resulted highly correlated to presence of yeasts and molds in silage, as well as to concentrations of mycotoxins produced by *A. fumigatus*, *P. roqueforti*, *P. paneum* and *Fusarium* spp. Anyway, if a mycotoxin contaminated forage is used, it should be recommended to discard moldy parts or any material that is contaminated by mycotoxins, to reduce its use in diets by substituting it with other available forages or fibrous by-products and to use adequate sorbent materials, as successively discussed. After economical and management evaluations, feeds proved to be too dangerous for animal health should not be used.

About ensiling methods, bunkers represent the most common system to ensiled crops, but also other methods are currently available, such as pile, silo bags, wrapped bales and tower silos [28,69]. For MS, González Pereyra *et al.* [302] reported occurrences of *Aspergillus* spp. and *Fusarium* spp. were higher in bunker silos, whereas *Penicillium* spp. incidence was higher in silo bags. Contrarily, Gallo *et al.* [69] did not report an effect of adopted ensiling procedures on contaminations of *Aspergillus*, *Penicillum* or *Fusarium* derived mycotoxins. Consequently, these aspects require further investigations.

### 4.2. Detoxification and Biodegradation of Mycotoxins on Farm Conditions

Because it is very difficult to prevent mycotoxin contamination either pre-harvest or during storage of feeds [4], several tools for neutralization of mycotoxins have been developed to protect animals from ingestion of contaminated feeds. Overall, the decontamination and detoxification procedures have to respect some guidelines [4,56,303]: be effective in the inactivation, destroy or removal of the mycotoxins; not result in the deposition of toxic or carcinogenic/mutagenic substances, metabolites or by-products in feeds and food; retain nutrient value and feed acceptability of the products or commodities; not result in significant alterations of the product’s technology properties; be economical and technologically convenient; not alter the cost of final product and destroy fungal spores to avoid a late contamination.

As recently reviewed, the inclusion of sorbent materials in animal diets or the addition of enzymes or microorganisms capable of detoxifying mycotoxins have been reported to be reliable methods for prevention of mycotoxicosis in farms [2,6,18,22,220,285,303,304,305,306,307]. In particular, mycotoxin sequestering agents are compounds able to bind mycotoxins in contaminated feeds without dissociating toxin-sequestering agent complex, thus it could pass through the gastrointestinal tract of animals and toxin could be eliminated via feces [29,303,308]. Many studies have been carried out on inorganic and organic binders and we refer to previous mentioned reviews for details. Among inorganic sequestering agents, clays are largely used as binding agents for reducing AFB_1_ intoxication of livestock and AFM_1_ carry over into milk for lactating animals [29,164,218,306,308,309,310,311,312]. In addition, organic sequestering agents, such as activated carbon and yeast cell wall products, have been reported to efficiently reduce AFM_1_ in milk of cows fed AFB_1_-contaminated feed [313,314], even if the efficacy to bind AFs of some yeast cell wall products is still controversially discussed [218,220]. Anyway, all *in vivo* studies, carried out on ruminants, tested the sequestering efficiency of different adsorbents against AFs [6] and some *Fusarium* derived toxins, such as DON, ZEA and 15-acetyl-deoxynivalenol [180,181]. Furthermore, a mycotoxin deactivating product was tested on lactating dairy cows fed diets naturally contaminated by AFs [315] or AFs and several *Fusarium* derived toxins, such as DON, ZEA, FB_1_, OTA and T-2 toxin [171] and now it is approved for its use in pig diet by European Union [18]. Its mode of action is based on three strategies [18,315,316]: (1) polar mycotoxins (e.g., AFs) are adsorbed by the inorganic components; (2) other mycotoxins not or poorly absorbed by the inorganic components (e.g., trichothecenes, ZEA) are biotransformed by biological constituents, namely, *Eubacterium* strain (BBSH 797) and a yeast strain (*Trichosporon mycotoxinivorans* MTV) able to alter mycotoxin structures into non-toxic metabolites which are excreted and (3) protective action against mycotoxins acts by mycotoxins phycophytic substances derived from a species of sea alga (*Ascophyllum nodosum*) and plant (*Silybum marianum*) extracts, as reported by Pietri *et al.* [315]. When supplemented to contaminated diets, the product reduced AFM_1_ extraction into milk without interfering with feed intake and milk production [315] or increase both milk yield and milk protein in cows fed multi-contaminated (both *Aspergillus* and *Fusarium* derived mycotoxins) diets [171]. Alternative to the use of sorbent materials in animal diets, vaccination strategies were recently explored to prevent negative effects of mycotoxin (*i.e.*, AFs) ingestion in lactating dairy cows [317] and heifers [17] or to reduce carryover of AFM1 into milk and cheese.

Some *in vitro* trials were carried out to verify the activity of different adsorbents for mycotoxins different from AFs, such as DON, ZEA and FBs [313,318,319,320,321,322]. To the best of our knowledge, no information are currently available about sequestering efficiency of different products against many *Alternaria*, *Aspergillus*, *Penicillum* and *Monascus* derived toxins that could be detected in forages. Only recently, Santos and Fink-Gremmels [16] observed on commercial farms that the dietary supplementation of a glucomannan mycotoxin absorbent agent resulted efficient to prevent mycotoxincosis in dairy cattle exposed to ingestion of moldy silages. Consequently, *in vitro* and *in vivo* experiments are necessary to verify the efficacy of different commercially available sequestering agents on these kind of mycotoxins. In addition, for many contaminated diets the challenge is from the possible co-occurrence of a high number of mycotoxins, so what is required is to standardize sampling procedures, to use specific methods of analysis able to detect hundreds of mycotoxins simultaneously, as well as their modified forms, and to adopt opportune strategies for successfully mitigating the different negative effects of the wide range of mycotoxins contaminating animal diets.

## 5. Conclusions

Grazed forage, hay or silages are often contaminated by a wide range of mycotoxins and other fungal exometabolites produced by molds able to infect crops at the pre-harvest stage, during prolonged wilting in bad weather conditions or in silos, piles and bags post-harvest. Despite an increased awareness of mycotoxin occurrences in silages and other forage crops, data are still limited, and thus unsuitable for properly assessing the risk of mycotoxin exposure in cattle and other ruminant species. Consequently, it should be strongly recommended to analyze forages not only for nutritive and fermentative characteristics, but also for mycotoxin contaminations, being this aspect strongly related to the safety use of a given forage in animal diet.

Cases of performance reduction, illness and other diseases have been often associated with ingestion of mycotoxin contaminated forages, but a direct link between ingestion of these mycotoxins, such as those produced by *Aspergillus fuminatus*, *Penicillium roqueforti*, *P. paneum* and other mycotoxigenic molds able to grow on silages and other forage crops, and animal intoxication events are rarely reported and are often unconfirmed. Indeed, these filamentous fungi can produce several exometabolites with antimicrobial and immunosuppressive properties that cause indirect and difficult to observe sub-acute symptoms, such as a reduction in the rumen functionality or an increase in susceptibility of animals to infections.

Consequently, certain scientific evidences regarding negative effects of mycotoxin ingestion on health status and performance of cattle is scarce and still need to be proven. The lack of unequivocal information regarding mycotoxin effects on ruminants should be also related to the complexity to plan specific animal trials since a multitude of confounding effects, such as toxin-, animal-, diet- or environmental-related factors, mycotoxin co-occurrence in feeds and presence of modified mycotoxins, exist. Alternatively, modeling the mycotoxin effects in animals would be a worthwhile approach able to provide useful information and to identify critical research areas that should be investigated.

To prevent or, at least, counteract the negative effects of mycotoxin ingestion in cattle, farmers should adopt the best practices to grow and harvest crops in field, to store hay and grains before feeding or to ensile forages with the aim to reduce the zone exposed to risk of air penetration and aerobic instability. In particular, for many secondary metabolites produced by mycotoxigenic fungi usually detected in silage and other forage crops, few information are currently available about effectiveness of dietary supplementation of adsorbent materials. There is a need to carry out specific trials for investigating sequestering efficiency of different adsorbent products against many *Alternaria*, *Aspergillus*, *Penicillium* and *Monascus* derived toxins that are normally detected in forages.

Last, in order to provide information concerning risk of mycotoxin contamination in cattle diets and to verify the mycotoxin ingestion in animals, an international network including participants of each ring of the food chain should be created to monitor mycotoxin occurrence in silages and other forage crops, thus permitting to verify exposition levels to mycotoxin ingestion of ruminants. Furthermore, shared *in vitro* or *in vivo* trial protocols are strongly preferable to standardize methodology and data interpretation.
